# A three-way comparative genomic analysis of *Mannheimia haemolytica *isolates

**DOI:** 10.1186/1471-2164-11-535

**Published:** 2010-10-04

**Authors:** Paulraj K Lawrence, Weerayuth Kittichotirat, Jason E McDermott, Roger E Bumgarner

**Affiliations:** 1 Department of Veterinary Microbiology and Pathology, Washington State University, Pullman, WA 99164-7040, USA; 2 Department of Microbiology, University of Washington, Seattle, WA 98195-7242, USA; 3 Pacific Northwest National Laboratory, Richland, WA, 99352, USA

## Abstract

**Background:**

*Mannhemia haemolytica *is a Gram-negative bacterium and the principal etiological agent associated with bovine respiratory disease complex. They transform from a benign commensal to a deadly pathogen, during stress such as viral infection and transportation to feedlots and cause acute pleuropneumonia commonly known as shipping fever. The U.S beef industry alone loses more than one billion dollars annually due to shipping fever. Despite its enormous economic importance there are no specific and accurate genetic markers, which will aid in understanding the pathogenesis and epidemiology of *M. haemolytica *at molecular level and assist in devising an effective control strategy.

**Description:**

During our comparative genomic sequence analysis of three *Mannheimia haemolytica *isolates, we identified a number of genes that are unique to each strain. These genes are "high value targets" for future studies that attempt to correlate the variable gene pool with phenotype. We also identified a number of high confidence single nucleotide polymorphisms (hcSNPs) spread throughout the genome and focused on non-synonymous SNPs in known virulence genes. These SNPs will be used to design new hcSNP arrays to study variation across strains, and will potentially aid in understanding gene regulation and the mode of action of various virulence factors.

**Conclusions:**

During our analysis we identified previously unknown possible type III secretion effector proteins, clustered regularly interspaced short palindromic repeats (CRISPR) and CRISPR-associated sequences (Cas). The presence of CRISPR regions is indicative of likely co-evolution with an associated phage. If proven functional, the presence of a type III secretion system in *M. haemolytica *will help us re-evaluate our approach to study host-pathogen interactions. We also identified various adhesins containing immuno-dominant domains, which may interfere with host-innate immunity and which could potentially serve as effective vaccine candidates.

## Background

*Mannhemia haemolytica *is a weakly haemolytic, Gram-negative bacterium and the principal casual agent associated with the respiratory-disease complex in ruminants. *M. haemolytica *is a normal commensal of the upper respiratory tract and tonsillar crypts in healthy ruminants. However, in the case of animals with compromised pulmonary defense mechanisms and stress, it can migrate into the lungs and cause acute fibrinous pleuropneumonia or pasteurellosis, commonly known as "shipping fever" [1-3]. Young animals are more susceptible than adults leading to sudden death with or without clinical signs [4]. Outbreaks of Pasteurellosis caused by *M. haemolytica *result in substantial economic losses to the global cattle industry and accounts for 30% of the total cattle deaths worldwide [5-7]. This impact is particularly devastating to the North American cattle and sheep industries [5,6]. The U.S beef industry alone loses more than one billion dollars annually to shipping fever [8]. In addition, *M. haemolytica *infection results in collateral losses to other domestic and wild ruminants.

Despite its enormous economic importance, there are no specific and accurate genetic markers to precisely understand the pathogenesis and epidemiology of *M. haemolytica *at molecular level. Commonly used genotyping techniques such as16 S rRNA sequence phylogeny, DNA:DNA hybridization, pulse field gel electrophoresis and restriction fragment length polymorphisms are unreliable, time consuming and cannot be correlated to pathogenesis or species specificity of an isolate [9-11]. The molecular basis of virulence mechanisms of *M. haemolytica *is fragmentary, due to the complex gene regulatory machinery involved during the expression of virulence and virulence-associated factors in host tissues [12]. In addition, until our recent publication of the genome sequences of two *M. haemolytica *serotype A2 strains, only one genome sequence from *M. haemolytica*, serotype A1 was available in the GenBank [13,14]. As a result, there was clearly a dearth of knowledge about the range and diversity of potential virulence factors in different strains of *M. haemolytica*.

The current classification of *M. haemolytica *relies on serotyping based on external capsular polysaccharides and twelve different serotypes have been identified. Furthermore, these serotypes do not conform to the classical Koch's postulates on microbial pathogenesis due to a great deal of genome plasticity and frequent serotype switching [15]. Of these, A1 and A2 are the most prevalent serotypes and are normal residents of the upper respiratory tracts of healthy cattle and sheep worldwide [16,17]. They are generally, but not exclusively, species-specific in their ability to cause pneumonia [16,17]. Bovine pneumonic pasteurellosis is mostly caused by *M. haemolytica *serotype A1, while the serotype A2 causes pneumonia in sheep. With the objectives of identifying the species specificity of serotypes at the molecular level, single nucleotide polymorphisms (SNPs) that may be associated with fibronecrotizing pneumonia, and identifying additional virulence factors, we sequenced two *M. haemolytica *A2 serotypes from two different ruminant species. One was isolated from the pneumonic lungs of domestic sheep (*Ovis aries*) and designated as Ovine (O), while the other isolate from cattle (*Bos taurus*), was designated as Bovine (B). We performed a genome-wide comparative sequence analysis between these strains and the genome sequence of *M. haemolytica *bovine serotype A1, PHL213 which we designated as A1.

During this investigation, we identified all the previously described virulence factors including lipopolysaccharide (LPS) biosynthesis, iron acquisition, complex carbohydrate biosynthesis, capsular polysaccharides and adhesion biosynthesis genes in both the B and O genomes. We used our data to identify genes that are unique to each strain as well as SNP variation between the strains with a focus on non-synonymous SNPs in known virulence factors. In pathogenic bacteria, SNPs serve as evolutionary markers and those present in virulence factors may aid in defining the host specificity at molecular level [18]. We identified clustered regularly interspaced short palindromic repeats (CRISPR), CRISPR-associated sequences (Cas) and a previously undetected possible type III effector protein secretory pathway, which may be implicated in modulating *M. haemolytica *pathogenesis in a species-specific manner. We identified high confidence SNPs (hcSNPs) within the leukotoxin (Lkt) operon across all three isolates but only one hcSNP between bovine isolates in the open reading frames (ORFs) encoding O-sialoendoglycopeptidase, a key enzyme which determines the host-specific colonization of the bacteria. Along with these genotypic markers, we identified various adhesins containing hedgehog/intein and hep/hag immuno-dominant domains, which could potentially be used to engineer vaccine strains.

## Construction and content

### Genome sequencing and assembly

*M. haemolytica *serotype A2 isolated from pneumonic lungs of domestic sheep (*Ovis aries*) and cattle (*Bos taurus*), were grown overnight in brain heart infusion broth at 37°C/200 rpm. The next day, cells were harvested and the total genomic DNA was extracted using QuickExtract™ Bacterial DNA Extraction Kit (Epicenter Biotechnologies) following the manufacturer's instructions. Genomic libraries for sequencing were prepared from 5 μg of total genomic DNA. The sequencing reaction was performed using the 454 pyrosequencing technology and run on Genome Sequencer FLX Instrument (Software 1.0.53) following the manufacturer's instructions (Hoffmann-La Roche Ltd) [19]. The raw data was assembled using the Newbler Assembler Software (Genome Sequencer 20, Version 1.0.53), with default parameters.

### Gene prediction and annotation

The assembled contig sequences were processed by our in-house pipeline for gene prediction and annotation using the genome of serotype A1 as a guide. We adapted the protocol previously developed by The Institute for Genomic Research (J. Craig Venter Institute). Briefly, Glimmer3, Exonerate and tRNAscan SE tools were used to predict protein-, rRNA- and tRNA-coding genes respectively [20-22]. All protein-coding genes were then annotated by using the RAST annotation pipeline http://rast.nmpdr.org/[23]. Each protein sequence was also BLAST searched against Clusters of Orthologous Groups of proteins (COGs) database using the NCBI BLAST package [24,25]. Then a COG identification number was assigned to each gene if the best BLASTP hit exhibits at least 80% sequence coverage in both query and hit sequences and at least 30% protein sequence identity. Finally, protein-coding genes were analyzed to identify putative frameshift mutations using BLAST Extend-Repraze http://ber.sourceforge.net/. Genes containing frameshift mutations were considered as putative pseudogenes. Unique genes of each genome were identified by BLAST searching each gene sequence to the genome of another strain with an E-value cutoff of 1e-6. A gene was considered unique if no significant hit was reported. Novel computational methods were used to detect evidence of secreted effector proteins for the type III secretion system and web based software (http://www.genome.jp/, KEGG pathway) was used to detect secretion system components [26].

### Identification of high confidence single nucleotide polymorphisms (hcSNPs)

We developed a protocol that utilizes raw 454 read data (Hoffmann-La Roche Ltd) to identify hcSNPs between two genomes. Given two sets of 454 reads (readA and readB) from different genomes, we first used the newbler assembler to create two independent sets of contigs, named as contigA and contigB. The contig sequence from one genome was used as a reference while the reads from the other genome were aligned based on them using gsMapper software. Specifically, two gsMapper runs were carried out, where readA was mapped to contigB in one run and readB to contigA in another run. The gsMapper software utilizes a reference sequence to aid the assembly of raw read data. In addition to sequence assembly, this software gives a "high confidence difference (HCD)" file that summarizes all regions, where the sequence alignment shows differences between reference and multiple read sequences spanning that region. From "readA mapped to contigB" gsMapper run, readA sequences spanning a HCD region (*e.g*., HCD1) were checked to identify regions within contigA sequence, where they were assembled by the previous newbler run. If this particular region also appears in the HCD output file from the "readB mapped to contigA" gsMapper run, where readB sequences spanning this HCD have been assembled to the region where the previous HCD1 is found, we considered this HCD to be an hcSNP. This protocol essentially allows us to screen for differences between two genomes that were supported by multiple raw sequencing reads. For the comparison of B and O strains, each hcSNPs are supported by at least 10 reads from each genome, where at least 80% of the reads spanning that region show the difference. For comparison of B or O to previously sequenced A1 serotype, we were limited by the lack of raw data from A1. Hence, we were able to compare only to the consensus sequence of A1 as reported in the GenBank. As a result, hcSNPs from comparisons to A1 are only supported by multiple reads from either B or O genome. We anticipate that the error rate in such an assembly will range from 1 part in 10,000 to about 1 part in 40,000. For a genome of approximately 2.2 Mb in length, we anticipate approximately 55-220 sequence errors in the assembly. Hence sequence differences between B/O and A1 will be a combination of both real sequence differences and errors in the assembly of A1. Finally, the hcSNP data is filtered based on contig base quality score (at least 60) as well as whether or not the hcSNP is found on a homopolymer region of 3 bases or more to reduce false positive error.

### Phylogenetic trees construction

We identified conserved genes common in all nine genomes based on their DNA sequence similarity using a combination of BLASTCLUST and BLAST software [25]. This resulted in a list of 28 genes, most of which coded for 30 S and 50 S ribosomal subunit proteins. The concatenated sequences of these 28 conserved genes (average length of 17,638 bp) were then aligned using ClustalW version 2 with default parameters [27]. PHYLIP program version 3.6 was used to construct the tree using the F84 evolutionary model and neighbor-joining method http://evolution.genetics.washington.edu/phylip/getme.html[28]. Finally, the Phylodendron software was used to draw the tree [29].

## Utility and discussion

### Gene content analysis

**Figure 1:**
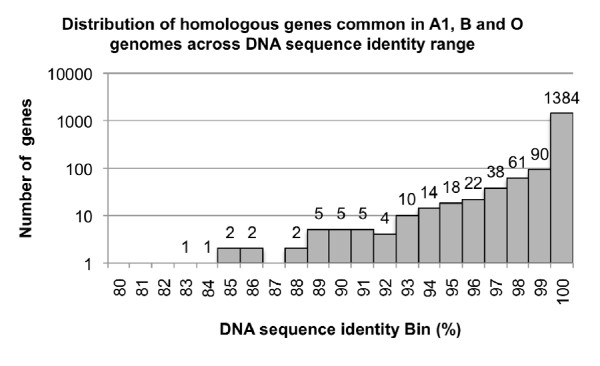
**Distribution of homologous genes that is common in A1, B and O genomes across DNA sequence identity range**. DNA sequences of 1,664 homologous protein-coding genes with length of at least 300 bp are considered in this analysis. This figure shows that most of the coding genes are highly conserved between across 3 strains *Mannheimia haemolytica *with an average DNA sequence identity of 99.2%.

We obtained 20× coverage for B and O genomes after high-throughput sequencing using the 454- pyrosequencing technology. The assemblies produced 84 contigs for the B (average contig length of 30.2 kb) and 144 contigs for the O (average contig length of 17.9 kb) genomes respectively. The average contig length selected for cut off was >300 bp. Based on our previous experience, at this coverage, the gaps in the sequence are likely to be small and typically associated with repeat regions. The total number of base pairs in the contigs were 2,478,004 for B and 2,584,200 for O, which is comparable to the only other previously sequenced *M. haemolytica *A1 draft genome with a 8× coverage (2,569,125 bp) [14]. The complete annotation of B and O strains with additional details can be found in the GenBank (accession numbers ACZY00000000 and ACZX00000000, project numbers 40173 and 40171 respectively) [13]. The overall GC content of the *M. haemolytica *genomes is approximately 41%. Using an automated gene finding and annotation pipeline (see construction and content), we identified 2,550 open reading frames (ORFs) in the B and 2,682 ORFs in the O genome. The number of genes identified in these genomes are comparable to the previously reported *M. haemolytica *A1 genome (2,695 ORFs, GenBank accession number AASA00000000). Table 1 summarizes the basic features of the B and O genomes. We found 82-100% (average = 99.2%) overall nucleotide identity among the 1,664 protein encoding genes that are at least 300 bp in length in all 3 strains (Figure 1), which suggests that most of these common genes are highly conserved among these three isolates.

**Table 1:** Basic features of *Mannheimia haemolytica *B and O genomes.

Strain	B	O
**Total length (Mb)**	2,478,004	2,584,200

**Number of genes with assigned function**	1,649	1,717

**Unassigned function**	538	575

**Putative pseudogenes**	151	160

**Transposase genes**	21	21

**Phage genes**	191	209

**Gene density**	89.2	87.9

**Average coding sequence size (bp)**	866	847

**Number of contigs**	84	114

**Average bp per contig**	30,219	17,945

**Number of coding sequences**	2,550	2,682

**Number of tRNA genes**	48	49

By performing an all genes against all genomes analysis, we found that the A1, B and O genomes have 233, 38 and 62 unique genes respectively (a full list of A1, B and O strain specific genes can be found in the Additional file 1, Table S1, Additional file 2, Table S2 and Additional file 3, Table S3 respectively). Among the A1 specific genes 57% are hypothetical proteins and 20% are bacteriophage encoded proteins. The A1 genome has a few functionally important enzymes such as the UDP N acetylglucosamine 2 epimerase (MHA_0505), capsule biosynthesis protein (MHA_0507) and sialyltransferase (MHA_1780), indicating their possible role in conferring serotype specificity. Similarly, 57% of the B genome specific genes are hypothetical proteins and 29% are phage proteins. Among the B specific genes, enzymes such as peptidoglycan transglycolyase (COK_0539) involved in peptidoglycan biosynthesis and beta hexosamidase (COK_2260), a glycoside hydrolase involved in trimming carbohydrate decorations and pathogenesis are unique and noteworthy. On the other hand, the O genome accounts for 70% hypothetical proteins, of which less than 2% originate from phages. The only specific protein in the O genome, is the silent information regulator (Sir2, COI_1007), a homolog of transcription regulator, which is critical in maintaining bacterial replication and gene expression.

**Figure 2:**
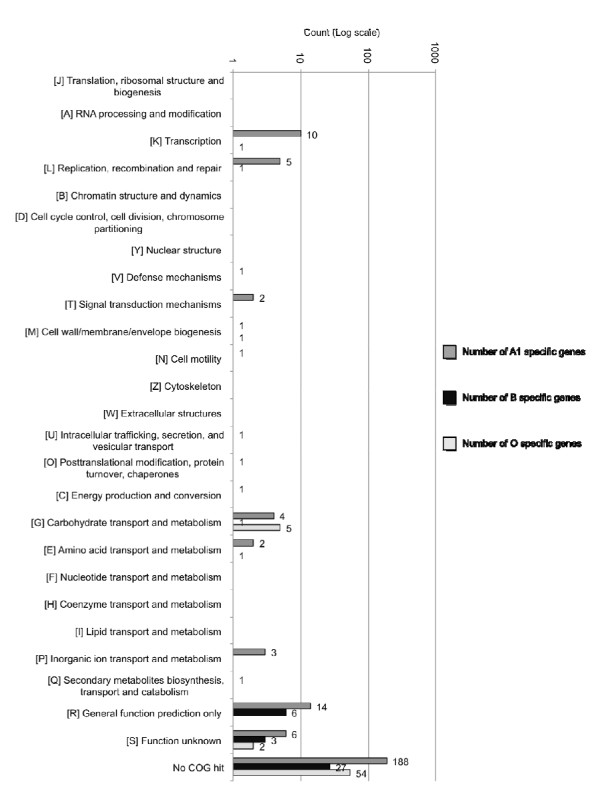
**Strain specific gene in each Clusters of Orthologous Groups, COG category**.

In summary, we found that the vast majority of these strain specific genes do not exhibit significant sequence similarity to other proteins of known function and cannot be categorized into any Clusters of Orthologous Groups (COG, Figure 2). However, the B and O strain specific genes appear to resemble integrated phage genomes. The pair wise comparison of gene content from one serotype in relation to the other reveals that about 9-12% of the genes are variable between A1 and A2 (B and O). Comparatively, a smaller number of variable genes (2-6%) are found between B and O, indicating that these two genomes share a more similar gene content in relation to each other than to A1 (Table 2). This degree of strain specificity and variable gene pool is an important group for further study, since bacteria frequently acquire virulence factors *via *horizontal gene transfer [30].

**Table 2:** Percentage of coding genes that are found in one genome but not the other.

Strain (# of genes/strain)	A1	B	O
**A1 (2692)**	0	12.1%	8.6%

**B (2550)**	9.2%	0	2.0%

**O (2682)**	9.6%	6.2%	0

### SNPs among virulence genes

Pathogenicity of *M. haemolytica *is due to a repertoire of exotoxins, endotoxins, and host immune-modulating proteins produced by it. Therefore, to characterize the potentially interesting SNPs in the *M. haemolytica *genome, we performed a SNPs analysis between these three genomes. In brief, we mapped individual reads from each strain to the consensus sequence of the other strains and identified SNPs that meet certain quality criteria (see construction and content). These SNPs are dubbed as high confidence single nucleotide polymorphisms (hcSNPs). The availability of individual reads from the strains of interest allowed us to compare the B and O genomes in both the directions (*e.g*., reads from B mapped to the consensus sequence of O and *vice versa*), which enabled us to filter the results and identify hcSNPs that meet the quality criteria for both mapping directions. These hcSNP's have high sequence coverage in both strains and consistently vary between strains in the individual reads.

To estimate the rate of false positives in our SNP detection methods and to obtain a quantitative estimate of the overall error of 454 sequencing and assembly, we have done the following: 1) We have previously sequenced two strains of *Pseudomonas aeruginosa *(unpublished data) to a very high depth of coverage (approximately 40×). 2) To estimate the precision in independently sequenced and assembled genomes, we have subsampled (without replacement), the reads from one these strains to create independent assemblies at 20× coverage (see Additional file 4, Table S4). 3) Assembled these sampled reads using the newbler assembler and mapped the trimmed contigs from these two independent assemblies onto each other to determine the precision, *e.g*., the differences that one obtains through sequencing and assembly errors when sequencing the same DNA. 4) Our results show that the difference between the two independent sequencing and assemblies was 1 bp in 17,681 bp (a total of 368 differences in a total of 6,506,615 aligned basepairs) and most (280 out of 368 or 76%) of these differences is in areas of homopolymer repeats. Since the B and O *M. haemolytica *genomes were sequenced to a similar depth (about 20×), we estimate a similar level of precision in the assemblies of the O and B genomes, *e.g*., approximately 1 difference in 17,681 bp due to sequencing and assembly errors. Furthermore, we estimate the precision in non-homopolymer repeat regions to be approximately 1 bp in 74 kb. The SNP's that we have called between the O and B genomes occur at a rate of approximately 1SNP per 646 bp. Since we have filtered out SNP's in homopolymer regions, an estimate of the false positive rate in our SNP calls is of order 1% (646 bp/74 kb = 0.008).

For the SNP comparisons between the O/B genomes and the A1 genome it is a bit more difficult to estimate the false positive rate, since we don't have the raw read data that went into the assembly of A1. However, we do have quality value estimates for each position in the assembly ftp://ftp.hgsc.bcm.tmc.edu/pub/data/MhaemolyticaPHL213/MhaemPHL213-10Aug2006-scaffolds.qual and we filtered SNPs to eliminate regions in the A1 assembly that are of Q-value <60. Even with this stringent filtering, we identified 13,705 and 9,968 hcSNPs between B and A1 and O and A1 respectively (or 1SNP/180 bp for B/A1 and 1SNP/259 bp for O/A1). Given that typical precision rates for assemblies from eight fold Sanger sequencing data are of order 1 bp in 20,000-100,000 bp, we assume that the A1 assembly is accurate to at least 1 bp in 20,000 [31]. Hence, we estimate that the rate of false positive for SNP detection in comparing the O/B genomes to the A1 genome is also of order 1%.

Table 3 summarizes the total number of synonomous and non-synonomous hcSNPs across all three isolates with additional files 1, 2 and 3 showing individual hcSNP genes with substitutions across each genome. In the following sections, we discuss the sequence variations which we identified in known virulence and virulence associated factors.

**Table 3:** Summary of high confidence single nucleotide polymorphisms between B *versus *O, B *versus *A1 and O *versus *A1 genomes

	B vs O	B vs A1	O vs A1
**Total hcSNP**	3,031	13,705	9,968

In coding region	2,860	12,901	9,403
- Synonymous	2,008	8,893	6,440
- Non-synonymous	823	3,753	2,697
- Indel	29	255	266

In non-coding region	171	804	565
- Substitution	156	694	498
- Indel	15	110	67

### Leukotoxin hcSNPs and sequence variation

**Figure 3:**
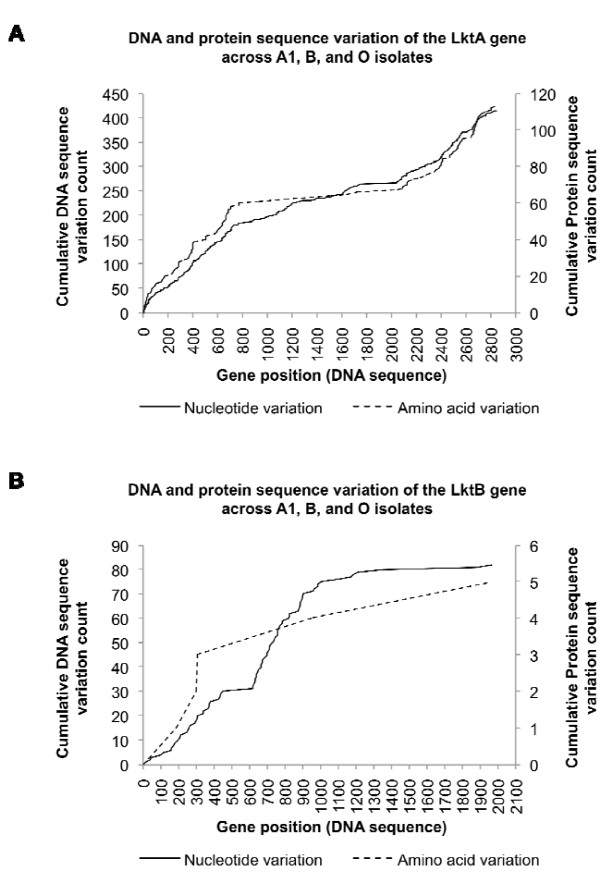
**Cumulative counts of nucleotide and amino acid variation of LktA (A) and LktB (B) genes across A1, B and O isolates**.

The leukotoxin (Lkt) secreted by *M. haemolytica *is a well studied, chief virulence factor [32-34]. Lkt is also produced by other members of gram-negative bacteria including *Aggregatibacter actinomycetemcomitans, Actinobacillus pleuropneumoniae *and *Escherichia coli *[35,36]. Lkt belongs to the RTX (repeats-in-toxin) family of pore-forming exotoxins. Although these toxins have broad target cell specificity, *M. haemolytica *Lkt is specific for ruminant macrophages, neutrophils and all leukocyte subsets [37]. The β2 integrins LFA-1, Mac-1 and CR4 expressed by ruminant polymorphonuclear leukocytes (PMNs), serve as receptors for *M. haemolytica *Lkt [38-42]. These receptors exhibit a high degree of plasticity by binding to Lkt produced by different *M. haemolytica *serotypes [40,41]. Furthermore, the leukotoxin operon (*lktCABD*) of *M. haemolytica *is a complex mosaic structure derived by extensive inter- and intra-species horizontal DNA transfer and intragenic recombination events [43]. LktA protein sequences of B, O and A1 (COK_0274, COI_0481 and MHA_0254 respectively) have an overall dissimilarity of 12%, accounting for 17% amino acid substitution at the amino- terminal (1-378) and 21% substitution at the carboxyl- terminal (780-953) (Figure 3A). However, the amino terminus of LktA of A1 has 51% amino acid substitution in this region when compared to B and O, but shares 98% overall identity with O and 88% with B respectively. The region encompassing the first 35 amino acids of LktA, which is involved in pore formation, is 100% identical between the O and B isolates [44]. At the nucleotide level, there are a total of 415 variations across the multiple sequence alignment of the *lktA *genes from B, O and A1 genomes, which account for 113 amino acid sequence variations (Figure 3A, Additional file 5, Figure S5). The multiple alignment result also shows that *lktA *sequences from O and A1 are more similar to one another and most of the variations identified by three-way comparisons are due to the *lktA *sequence of the B genome. Among all the variations identified, 116 mutations found between *ltkA *genes of B and O genomes are considered as hcSNP by our SNP filtering methodology encompassing 21 non-synonymous mutations (Additional file 6, Table S6). Similarly, 135 (28 non-synonymous) and 3 (1 non-synonymous) mutations found between B versus A1 and O versus A1 respectively are considered as hcSNPs (Additional file 7, Table S7, and Additional file 8, Table S8).

The DNA sequence of Lkt translocation ATP-binding gene, *lktB*, appears to be more conserved between the B, O and A1 genomes (COK_0273, COI_0482 and MHA_0255 respectively) relative to the *lktA *gene. The multiple sequence alignment shows 82 nucleotide substitutions, but amounts to only five amino acid substitutions (Figure 3B, Additional file 5, Figure, S5). The *lktB *sequence from A1 and O genomes are more similar to each other, but both are more diverged from the *lktB *sequence of the B genome. Out of the 54 mutations identified between *lktB *of B and O that are considered as hcSNP, five are non-synonymous (Additional file 6, Table S6). Similarly, out of the 78 mutations found between B and A1, four are non-synonymous and only one non-synonymous mutation is shared between O and A1 (Additional file 7, Table S7, and Additional file 8, Table S8). Although Lkt polymorphism has been implicated in species specificity, we found that the Lkt isolated from individual *M. haemolytica *isolates bind to β2 integrins from various ruminant species [40,41,43]. We suspect that this degree of polymorphism in LktA is important to enhance the ability of *M. haemolytica *serotypes to adapt to its niche, the ruminant respiratory tract.

Although Lkt is an important virulence factor, our earlier experiments have shown that *lktA*-deletion mutants of *M. haemolytica *still cause mild lung lesions with reduced mortality when compared to the wild type bacteria [45]. Therefore, it is logical to hypothesize that this organism has an arsenal of accessory virulence factors, which aid in host colonization, help gain a competitive advantage to act synergistically and modulate host gene expression. These accessory factors possibly include CRISPR and a type III secretion system identified during this study along with the previously known factors such as lipopolysaccharides, O-sialoglycoprotein endopeptidase, capsular polysaccharides, iron-regulated outer-membrane proteins and adhesins [46].

### Lipopolysaccharides

Lipopolysaccharide (LPS) is an integral part of the Gram-negative bacterial cell wall and is the chief endotoxin that contains pathogen-associated molecular patterns (PAMPs) [46]. LPS activates the macrophages through toll like receptors (TLRs) and elicits inflammatory cytokine production resulting in septicemia [46,47]. *M. haemolytica *LPS also induces an inflammatory cytokine response leading to increased expression of β2-integrins in the host [48]. The structure of *M. haemolytica *A1 O-antigen consists of trisaccharide repeat of two D-galactose residues and one N-acetyl-D-galactosamine residue [49]. The Leloir pathway catalyzes the epimerization of UDP-galactose to UDP-glucose in most Gram-negative bacteria, which is an important step in LPS biosynthesis [50]. This enzymatic reaction is carried out by UDP-galactose 4-epimerase (GalE). GalE mutants of *Salmonella enteric *serovar Typhimurium, *Neisseria gonorrhoeae*, and *Haemophilus influenzae*, which have truncated LPS, are avirulent when compared to their wild type, while in *M. haemolytica *they may abrogate adhesion [51-55]. Both the B and O genomes contain 32 out of the 38 previously reported genes found in the A1 LPS biosynthetic pathway (Additional file 9, Table S9). The A1 LPS biosynthetic pathway enzymes missing in the B and O genomes include UDP-N-acetylglucosamine 2-epimerase (MHA_0521), glycosyltranferase (MHA_1849, MHA_1850), possible sialyltransferase (MHA_1852) and hypothetical proteins (MHA_1847, MHA_1851). Most of the LPS biosynthetic genes shared between these three genomes exhibit 99-100% sequence identity (Additional file 9, Table S9), suggesting that the pathway leading to carbohydrate addition during LPS synthesis is critical and resistant to mutations.

### O-sialoglycoprotein endopeptidase

The adherence of pathogenic bacteria to mucosal epithelium is dependent on the expression of adhesive molecules or ligands called adhesions that allow attachment of the organisms to complementary molecules on mucosal surfaces or receptors. Pathogens from the family of *Pasteurellaceae*, employ various types of ligands, which enable them to adhere, colonize, and cause infection. These include pili, filamentous proteins (fimbriae), outer membrane proteins and capsular polysaccharides.

Although not cytotoxic, O-sialoglycoprotein endopeptidase helps *M. haemolytica *to colonize the upper respiratory tract of ruminants in a host-specific manner and serves as an important virulence factor. A1 and B are serotypically different, but the gene (MHA_1559 and COK_2067 respectively) encoding O-sialoglycoprotein endopeptidase has only one nucleotide substitution resulting in one amino acid substitution at position 70 (P → T) (Additional file 7, Table S7), while B (COK_2067) and O (COI_0128) show four substitutions, 70 (P → E), 191(T → E), 327 (S → G) and 340 (P → S) (Additional file 6, Table S6). Interestingly, the glycoprotease domain (amino acids, 96 - 116) remains conserved across all the three isolates.

### Iron-regulated outer-membrane proteins

Pathogenic bacteria often use iron as an environmental signal for the regulation of virulence genes [56,57]. In mammalian host, pathogens from the families of *Neisseriaceae *and *Pasteurellaceae *frequently deal with the lack of free iron for uptake, as most of it is stored in intracellular or extracellular (transferrin, lactoferrin, haemopexin and haptoglobin) bound forms [58,59]. To overcome this problem, Gram-negative pathogenic bacteria have evolved an elaborate iron-regulatory system, to acquire this element from the host. These include the direct binding of iron-containing proteins to outer-membrane receptors and the secretion of siderophores or haemophores [60]. Although *M. haemolytica *has no known siderophores, these bacteria produce two highly conserved transferrin (Tf)-binding proteins that specifically bind the Tf of their particular host [61,62]. Earlier experiments clearly indicate that iron is required for the proper growth and Lkt production in *M. haemolytica*, as pathogenic gonococcal mutants devoid of these proteins lose their virulence [63,64]. The B and O genomes encode several iron acquisition and iron homeostasis proteins similar to serotype A1. The presence of an elaborate set of iron-acquisition genes, therefore reiterates the importance of iron in controlling the transcription and expression of Lkt and other virulence factors in modulating *M. haemolytica *pathogenesis. Expression of these proteins allows *M. haemolytica *to acquire iron from host the hemoglobin, hemopexin, and transferrin. B and O encode two hemoglobin receptors HmbR1 (COK_2539 and COI_1763) and HmbR2 (COK_1624 and COI_2258) that are 99-100% identical to HmbR1 (MHA_1639) and HmbR2 (MHA_2261) of A1. The previously described transferrin-binding proteins, TbpA (MHA_0196) found in A1, is shared by B (COK_1753) and O (COI_2333) with 98% DNA sequence identity, but the TbpB (MHA_0197) is only 50% identical to TbpB, found in the B and O genomes (COK_1752 and COI_2332 respectively).

### Adhesins

Adhesins help in tethering the bacteria to the host cell surface in a manner similar to a grappling hook. Most of the adhesins are pili, and the type IV pilus locus *pilABCD *of O (COI_1201-COI_1998) and B (COK_1994-COK_1991) genomes are encoded in an opposite orientation, when compared to A1 (MHA_0662-MHA_0665). The PilC protein from A1 and B are 100% identical, indicating their bovine origin, whereas, strain O has a 29 amino acid deletion in its amino-terminus. PilC in *N. meningitides *is implicated in human epithelial cell-specific interaction and pilus biogenesis [65,66]. The amino-terminal deletion of PilC in *M. haemolytica *strain O may serve as a modification, necessary for ovine epithelial cell-specific colonization. The biogenesis and function of type IV pili is controlled by a large number of genes, almost 40 of which has been identified in *P. aeruginosa *[67]. B and O genomes share a high degree of homology to a number of genes required for pili assembly that are involved in type II protein secretion and competence for DNA uptake, suggesting that these systems share a common hierarchy along with *H. influenzae*, *P. aeruginosa*, and Neisseria species [68].

Filamentous hemagglutinin (FHA) is a major cell surface-associated adhesin that attaches to the host ciliary epithelial cells and is a virulence determinant [69,70]. Pfam http://pfam.sanger.ac.uk/ analysis indicates the possible involvement of internal FhaB domain of FHA protein in heme utilization. FHA (fhaB) from A1 (MHA_0866) shares 99% DNA sequence identity to the homologous regions of strains B (COK_0334) and O (Contig00015). The fhaB ortholog of *M. haemolytica *is also shared by *Bordella pertussis*, *N. meningitidis*, *A. pleuropneumoniae*, *M. succiniciproducens *and *P. syringae*. The fhaB genes of the B and O strains are adjacent to the fhaC ortholog, similar to the two-partner secretory pathway found in A1. The fhaB proteins in the B and O genomes lack the integrin-binding RGD motif, but are characterized by the presence of three bacterial intein-like (BIL) regions at their carboxyl- termini, similar to A1. BILs belong to the HINT (hedgehog/intein) superfamily of domains, which post-translationally self-process by protein splicing and self-cleavage, hence interferes with the host innate immune system [71].

The adhesin, serotype A1-specific antigen (Ssa1) is present in both the B and O genomes [72]. Surprisingly the amino acid sequence of Ssa1 from A1 (MHA_2492) is only 79% identical to B (COK_2411) considering their origin from a common host, but 95% identical to O (COI_0850). The Ssa1 protein also contains an amino-terminal peptidase S8 superfamily domain, which can be cleaved by serine peptidases and a carboxyl-terminal autotransporter superfamily domain.

The orthologs of *H. influenzae *and *N. meningitides *IgA-specific serine metallo-endopeptidase of A1 (MHA_0563 and MHA_2800), is 99% identical at amino acid level to B and O enzymes (COK_0634, COI2_430 and COK_1350, COI_2438). However, the MHA_2800 homologues of B and O (COK_1350, COI_2438) are only half the size and are devoid of amino acids ranging from 704-1503, including their carboxyl- terminal. This deletion removes the entire pertactin and autotransporter domains and almost 75% of the second peptidase S6 domain. These domains are not predicted to contain any active amino acids http://pfam.sanger.ac.uk/search/sequence. On the other hand MHA_0563 homologues, COK_0634 and COI_2430 contain all the three domains, *i.e*., S6 peptidase, AT-pertactin and carboxyl- terminal autotransporter. Iga1 hydrolyses the host mucosal antibody IgA and possibly IgG, and helps in colonization by immune-evasion [73].

The autotransporter/adhesion protein of A1 (MHA_2701), shares 75% identity with B (COK_1437) and O (COI_2393) genomes, whereas the AT family of autotransporter/adhesion MHA_1367 shares 96% (COK_2435) and 99% (COI_1943) identities respectively. These proteins are involved in promoting adhesion to the host mucosal surfaces and are closely related to autotransporter/adhesins of *A. pleuropneumoniae*, *M. succiniciproducens *and hep/hag family proteins of *N. mucosa*. The hep/hag domain is a seven-residue repeat that makes up the majority of the sequence of a family of bacterial haemagglutinins and invasins. The ORFs COK_2435 and COI_1943 show four and seven hep/hag repeats and one carboxyl-terminal YadA-like domain from *Yersinia *species. The hep/hag proteins also serve as immuno-dominant antigens in *Burkholderia mallei *and *B. pseudomallei *[74]. Therefore, hep/hag domains can be exploited for serodiagnosis in *M. haemolytica *along with hcSNP markers, for increased fidelity.

### Clustered regularly interspaced short palindromic repeats

**Figure 4:**
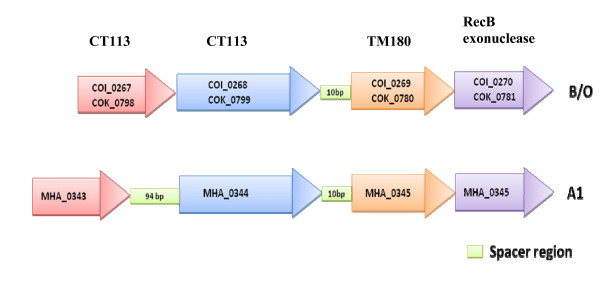
**Schematic representation of CRISPR/Cas locus in B, O (A) and A1 (B)**. All the three isolates have a 10 bp spacer region between CT1133 and TM1801 genes. The A1 also locus has an additional 94 bp spacer between CT1134 and CT1133 genes, which is absent in B and O genomes.

CRISPR loci consists of a family of DNA direct repeats separated by regularly sized non-repetitive spacer sequences that are found in most bacterial and archaeal genomes [75]. CRISPR regions provide acquired immunity against super infecting bacteriophages, possibly acting *via *RNA interference-like mechanism [76]. The differences in the number and type of the spacers between CRISPR repeats correlate with phage sensitivity. CRISPR regions are often hypervariable between the otherwise closely related strains [77]. In addition, there are many protein families known as CRISPR-associated sequences (Cas), which are encoded in the vicinity of CRISPR loci [78]. CRISPR/Cas gene regions can be quite large, with up to 20 different, tandem-arranged *cas *genes next to a CRISPR cluster or filling the region between two repeat clusters. Cas proteins are thought to be involved in the propagation and functioning of CRISPRs and some show similarity to helicases and repair proteins [78]. The CRISPR/Cas loci of B and O consist of four genes, COK_0798, COK_0799, COK_0800, COK_0801 and COI_0267, COI_0268, COI_0269, COI_0270 respectively. The Cas family, which is represented by CT1134 and CT1133 from *Chlorobium tepidum*, is also found in the B and O genomes (COK_0798, COK_0799 and COI_0267, COI_0268). This family belongs to the three-gene CRISPR/Cas subtypes found in *Desulfovibrio vulgaris *and is a member of the *Dvulg *subtype [78]. There are five hcSNPs in CT1134 and eleven in CT1133 loci between strains O and B, which can be used for strain typing. The locus, COK_0800 and COI_0269 are 100% identical to *Thermotoga maritima *Cas family, TM1801 [78]. The last gene in this cluster is a recB exonuclease (COK_0801 and COI_0270) which is 100% identical between these two isolates. Furthermore, we also identified CRISPR/Cas loci in the A1 genome (MHA_0343, MHA_0344, MHA_0345, and MHA_0346), previously reported as hypothetical proteins [14]. A schematic representation of the CRIPSR loci along with spacers from all the three isolates is shown in Figure 4. The presence of CRISPR regions in the *M. haemolytica *genome indicates its potential resistance to superinfection by phages. The CRISRP/Cas mechanism is analogous to the eukaryotic RNAi system [79,80].

### Type III secretion system

**Figure 5:**
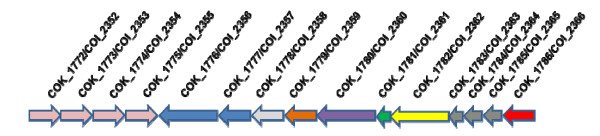
**Schematic representation of possible type III secretion system (T3SS) in B/O genomes**. COK_1772/COI_2352 to COK_1775/COI_2355- Putative arginine superfamily ATP binding cassette; COK_1776/COI_2356 & COK_1777/COI_2357- Cross over junction endonuclease Ruv A & B; COK_1778/COI_2358- RhtB family homoserine/threonine resistance; COK_1779/COI_2359- Acid phosphatase; COK_1780/COI_2360- Thiamine phosphate kinase; COK_1781/COI_2361- N utilization substance B; COK_1782/COI_2362- Type III secretion protein YscC homolog (competence protein E); COK_1783/COI_2363 to COK_1785/COI_2365- Hypothetical proteins, COK_1786/COI_2366- Putative pilus protein ComA.

We performed a novel computational analysis to search for previously undiscovered secretion systems in these genomes. In addition to genes encoding a type I secretion system similar to *E. coli *haemolysin, both the B and O genomes also contain genes that encode a previously undiscovered putative type III secretion system (T3SS) and twin arginine targeting (Tat) systems [81]. B and O encode proteins that show low to moderate homology to T3SS effector components of *E. coli *O157:H7, needle-like protein, SctF (49%), secretin SctC (35%), outer membrane protein SctW (40%), inner membrane proteins SctJ (37%), SctR (< 20%), SctS (> 20%), SctT (> 20%), SctU (41%), StcV (low), an ATPase SctN (46%) and an ATPase associated protein SctQ (low). They also share 40% identity to SctC and 47% to an ATPase SctN of *B. pseudomallei*. The genes encoding possible T3SS of B and O do not fall under a unique operon. The possible organization of T3SS genes is depicted in figure 5 along with type III secretion protein Ysc C homolog (COK_1782/COI_2362), which has a distinct secretin N-domain. Interestingly, we failed to identify T3SS operon in the A1 genome, due to either low sequence coverage or complete absence.

The presence of a T3SS in *M. haemolytica *B and O could equip them with a unique virulence mechanism that enables them to inject bacterial effector proteins directly into the host cell cytoplasm. The T3SS bypasses the extracellular milieu and potentially facilitates bacterial pathogenesis by specifically interfering with host cell signal transduction/transcription and other cellular processes [82,83]. Additional file 10, Table S10 describes the list of possible effector proteins showing high, moderate and low probability of being secreted through T3SS across all the three isolates. These effectors were predicted using a machine-learning method that uses the N-terminal region of proteins to predict secretion and does not rely on detecting homologous effector domains [26]. Almost 50% of the proteins predicted to be exported through T3SS are hypothetical and phage proteins. It is interesting to note that the Lkt acyl transferase and LktA are predicted to be highly exported through this machinery, although LktA has been shown to be secreted by type 1 system [84]. A family of metallo-endopeptidase S6, specific for cleaving IgA (COK_1350 and COI_2438) is also secreted with high probability. Similary, the list of moderately exported protein is also filled with a large number of hypothetical and phage proteins. There are quite a few virulence associated proteins, that are exported with moderate probability, and includes a S6 metalloendopeptidase homolog, (COK_0634 and COI_2430), iron scavenging transferrin binding protein (COK_1753 and COI_2333), siderophore esterase (COK_ 1515 and COI_0656), TonB (COK_1730, COI_2309), which is required to transduce cytoplasmic membrane energy to the outer membrane, Ton B dependent outer membrane receptor (COK_0223 and COI_0091), hemoglobin receptor (COK_2540 and COI_1762) and a neuraminidase (COK_1504 and COI_0667). Secretion of these proteins clearly indicates that *M. haemolytica *interacts with the host immune system, by bypassing mucosal defense and by scavenging iron to colonize.

One of the interesting effector protein from B, O and A1 (COK_1445, COI_1309 and MHA_0531) shows 20-40% identity to a transcription activator-like (TAL) type III effector, gene locus from *Xanthomonas oryzae *(AvrXa27), that activates the transcription of the host resistance gene *Xa27*, resulting in resistance to bacterial blight in rice [85]. TAL effectors target host general transcription factors and manipulate the host transcriptional machinery for virulence and/or avirulence [86]. To date, no such effector proteins have been identified from any *Mannheimia *species, which re-route the ruminant transcriptional/translational machinery to their advantage. Further experimental evidence directed towards isolating and characterizing these effector molecules from *M. haemolytica *will be necessary to confirm these computational inferences.

The twin arginine translocation (Tat) machinery exports folded proteins across the cytoplasmic membrane. Many Tat-secreted proteins are periplasmic enzymes that catalyze multiprotein oxido-reduction systems involved in respiration or anaerobic growth [86]. *M. haemolytica *Tat proteins ABC and E shares high homology with *E. coli *O157:H7 sec-independent translocase components (43%, 41%, 65% and 53% respectively). The presence of Tat pathway in *M. haemolytica *is interesting, because all proteins or complexes of proteins destined for Tat export must be covalently attached to one of these specialized amino-terminal twin-arginine signal peptides unlike the Sec pathway [87]. The Tat system is also an important virulence factor in aiding bacterial pathogens to infect plants and animals [88,89]. The B and O genomes encode trimethylamine *N*-oxide (TMAO)-inducible operon (*torABCE*), which is orthologous to *E. coli *[90]. The TorA protein, trimethylamine N-oxide reductase relies heavily on the bacterial twin-arginine system for its export [87]. Various bacteria grow anaerobically using TMAO as an alternative terminal electron acceptor of a respiratory transport chain [91], but its presence in *M. haemolytica *is intriguing since this organism grows in an oxygen rich environment.

### Phylogenetic analysis

**Figure 6:**
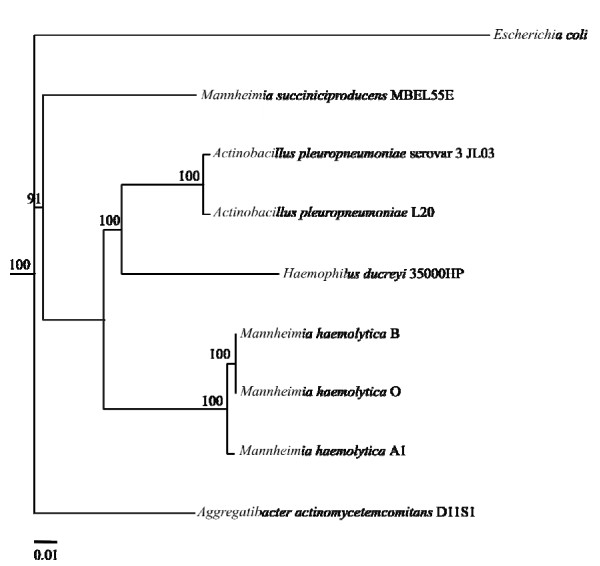
**Phylogenetic tree derived from concatenated sequences of 28 conserved genes**. Bootstrap values (100 replicates) are given at branch points as described in the methods section. Bar represent 0.01 substitutions per site.

Overall, the phylogenetic tree (Figure 6) constructed in this study is in congruence with the earlier results indicating that *M. haemolytica *B, O, A1, *A. pleuropneumoniae*, and *H. ducreyi *form a group that is divergent from the other members of the family, *Pasteurellaceae *[14]. Due to the limitations in using 16 S rRNA gene sequences for phylogenetic analysis, especially in the *Pasteurellaceae *species [14,92], we used genes encoding various 30 S and 50 S ribosomal subunits. In this analysis, the *M. haemolytica *isolates are closely related to *H. ducreyi *followed by two different isolates of *Actinobacillus*. This is in agreement to the earlier analysis obtained using the housekeeping genes from A1 [14]. *A. pleuropneumoniae *and *M. haemolytica *occupy the upper respiratory tract of their hosts and operate using identical virulence mechanisms and transition from commensalism to pathogenesis. On the other hand, *H. ducreyi *is an opportunistic organism that infects human genitalia through breaks in the skin or epidermis and appears to be closely related to *M. haemolytica *based on its competence genes, but shares the lowest number of orthologs with B, O and A1 [14]. Based on these analyses, it is reasonable to expect that *M. haemolytica*, *H. ducreyi *and *A. pleuropneumoniae *share a common *Pasteurellaceae *ancestory when compared to *M. succiniciproducens *which had been clustered with *Mannheimia *species based on 16 S rRNA sequences [92]. *M. succiniciproducens *MBEL55E used in this analysis is a capnophilic ruminant rumen bacterium, thus it is expected to have a low similarity to the other members of *M. haemolytica *cluster. Furthermore, it lacks virulence-related genes such as the leukotoxin and capsule biosynthesis genes [93]. Considering these phylogenetic trees, it will be interesting to see how the taxonomic classification of other *M. haemolytica *isolates fall into, once their genome sequences become available. Furthermore, comparative genomic sequence analysis involving other RTX toxin secreting pathogens will help in analyzing their molecular drives in causing such divergence.

## Conclusions

In summary, we have performed a three-way comparison between the genomic sequences of three strains of *M. haemolytica*. We identified a number of genes that are unique to each strain, which are "high value targets" for future studies that attempt to correlate the variable gene pool with phenotype (strain virulence, species specificity, etc). We also identified a number of hcSNPs and focused on non-synonymous SNPs in known virulence genes. This data will be used to design new hcSNP arrays that will aid in studying variation across strains and will potentially aid in understanding gene regulation and the mode of action of various virulence factors. The additional virulence factors identified in this study include a previously unknown type III secretion system and CRISPR regions, which can modulate host immune responses. These additional virulence factors along with adhesins, which contain protease cleavage domain will be used to investigate bovine immune responses and will serve as effective candidates for vaccine development.

## Availability and requirements

The B and O genomes can be accessed *via *NCBI database through the following URLs: http://www.ncbi.nlm.nih.gov/sites/entrez?db=genome&cmd=Retrieve&dopt=Overview&list_uids=6714 (B) and http://www.ncbi.nlm.nih.gov/sites/entrez?db=genome&cmd=Retrieve&dopt=Overview&list_uids=6715 (O) respectively.

## Abbreviations

SNPs: Single nucleotide polymorphisms; bp: base pairs; kb: kilo bases; Mb: mega bases: ATP: Adenosine Tri Phosphate.

## Authors' contributions

PKL and REB conceived and formulated the MS outline. PKL wrote the major part of the manuscript. PKL, REB and WK analyzed the data; WK produced all the figures and tables. JEM used computational software to analyze T3SS and effectors. All authors read and approved the final manuscript.

## Supplementary Material

Additional file 1 **Table S1**: *M. haemolytica *A1 specific genes. Click here for file

Additional file 2 **Table S2**: *M. haemolytica *Bovine (B) specific genes. Click here for file

Additional file 3 **Table S3**: *M. haemolytica *Ovine (O) specific genes. Click here for file

Additional file 4 **Table S4**: Summary of data used for precision analysis and precision statistics. Click here for file

Additional file 5 **Figure S5**: Multiple sequence alignment of LktA DNA and protein sequences. Click here for file

Additional file 6 **Table S6**: B vs O, high confidence SNPs. Click here for file

Additional file 7 **Table S7**: B vs A1, high confidence SNPs. Click here for file

Additional file 8 **Table S8**: O vs A1, high confidence SNPs. Click here for file

Additional file 9 **Table S9**: *M. haemolytica *LPS and other complex carbohydrate synthesis components. Click here for file

Additional file 10 **Table S10**: Type 3 effectors proteins from B, O and A1. Click here for file
