# Supplementary material for: A three-way comparative genomic analysis of Mannheimia haemolytica isolates
Source: BMC Genomics. 2010 Oct 4;11:535. doi: 10.1186/1471-2164-11-535 (PMC3091684; doi:10.1186/1471-2164-11-535)
Supplement: Additional file 1 — Table S1: M. haemolytica A1 specific genes. [file 1471-2164-11-535-S1.DOC]

**Table S1**: *M. haemolytica* A1 specific genes

| **Genome ID** | **Gene ID** | **Start** | **Stop** | **%GC** | **COG** | **Product** |
| --- | --- | --- | --- | --- | --- | --- |
| Phl213 | MHA_0001 | 6 | 314 | 0.35 | uncategorized | hypothetical protein MHA_0001 |
| Phl213 | MHA_0002 | 1087 | 350 | 0.34 | uncategorized | hypothetical protein MHA_0002 |
| Phl213 | MHA_0003 | 1613 | 1239 | 0.34 | uncategorized | hypothetical protein MHA_0003 |
| Phl213 | MHA_0004 | 2281 | 1625 | 0.40 | COG1396 | possible LexA family repressor/S24 family protease |
| Phl213 | MHA_0010 | 5671 | 5232 | 0.40 | uncategorized | possible integrase/recombinase |
| Phl213 | MHA_0012 | 6930 | 7325 | 0.38 | uncategorized | possible bacteriophage tail protein |
| Phl213 | MHA_0014 | 8081 | 8482 | 0.44 | uncategorized | hypothetical protein MHA_0016 |
| Phl213 | MHA_0015 | 8527 | 8757 | 0.46 | uncategorized | hypothetical protein MHA_0017 |
| Phl213 | MHA_0016 | 8825 | 9091 | 0.33 | COG3077 | possible RelB protein |
| Phl213 | MHA_0018 | 9439 | 9702 | 0.30 | uncategorized | hypothetical protein MHA_0020 |
| Phl213 | MHA_0022 | 13764 | 13922 | 0.37 | uncategorized | hypothetical protein MHA_0027 |
| Phl213 | MHA_0023 | 14230 | 13931 | 0.40 | COG4683 | possible transcriptional regulator |
| Phl213 | MHA_0024 | 14591 | 14202 | 0.37 | COG4683 | hypothetical protein MHA_0029 |
| Phl213 | MHA_0040 | 27225 | 27440 | 0.45 | uncategorized | hypothetical protein MHA_0046 |
| Phl213 | MHA_0318 | 361731 | 361291 | 0.38 | COG1943 | transposase |
| Phl213 | MHA_0319 | 361814 | 362890 | 0.41 | COG0675 | transposase |
| Phl213 | MHA_0325 | 369535 | 370455 | 0.26 | uncategorized | hypothetical protein MHA_0347 |
| Phl213 | MHA_0326 | 370618 | 370749 | 0.23 | uncategorized | hypothetical protein MHA_0348 |
| Phl213 | MHA_0338 | 382790 | 382623 | 0.35 | uncategorized | hypothetical protein MHA_0360 |
| Phl213 | MHA_0341 | 388043 | 387438 | 0.34 | uncategorized | hypothetical protein MHA_0364 |
| Phl213 | MHA_0374 | 422525 | 422193 | 0.32 | uncategorized | hypothetical protein MHA_0398 |
| Phl213 | MHA_0391 | 432468 | 432121 | 0.31 | uncategorized | hypothetical protein MHA_0415 |
| Phl213 | MHA_0397 | 435833 | 435462 | 0.32 | uncategorized | hypothetical protein MHA_0421 |
| Phl213 | MHA_0398 | 436167 | 435997 | 0.33 | uncategorized | hypothetical protein MHA_0422 |
| Phl213 | MHA_0400 | 437890 | 437258 | 0.39 | COG4385 | bacteriophage tail formation protein I |
| Phl213 | MHA_0406 | 442941 | 443219 | 0.32 | uncategorized | hypothetical protein MHA_0430 |
| Phl213 | MHA_0455 | 506396 | 505635 | 0.38 | uncategorized | FadR family transcriptional regulator |
| Phl213 | MHA_0456 | 506561 | 506445 | 0.29 | uncategorized | hypothetical protein MHA_0478 |
| Phl213 | MHA_0457 | 506560 | 507585 | 0.38 | COG1063 | zinc (Zn2+)-dependent dehydrogenase |
| Phl213 | MHA_0458 | 507614 | 508597 | 0.35 | COG1638 | TRAP-T family tripartite ATP-independent periplasmic transporter, binding protein |
| Phl213 | MHA_0459 | 508658 | 509137 | 0.35 | COG3090 | TRAP-T family tripartite ATP-independent periplasmic transporter, membrane protein |
| Phl213 | MHA_0460 | 509140 | 510411 | 0.38 | COG1593 | TRAP-T family tripartite ATP-independent periplasmic transporter, membrane protein |
| Phl213 | MHA_0505 | 551130 | 552245 | 0.39 | COG0381 | UDP-N-acetylglucosamine 2-epimerase |
| Phl213 | MHA_0507 | 553535 | 555655 | 0.32 | uncategorized | possible capsule biosynthesis protein |
| Phl213 | MHA_0508 | 555657 | 556724 | 0.34 | uncategorized | hypothetical protein MHA_0524 |
| Phl213 | MHA_0509 | 556736 | 557542 | 0.33 | uncategorized | hypothetical protein MHA_0525 |
| Phl213 | MHA_0510 | 557544 | 558539 | 0.35 | uncategorized | hypothetical protein MHA_0526 |
| Phl213 | MHA_0550 | 598407 | 598177 | 0.36 | uncategorized | hypothetical protein MHA_0570 |
| Phl213 | MHA_0551 | 598514 | 598437 | 0.39 | uncategorized | hypothetical protein MHA_0571 |
| Phl213 | MHA_0552 | 598845 | 598528 | 0.34 | uncategorized | hypothetical protein MHA_0572 |
| Phl213 | MHA_0553 | 598996 | 598838 | 0.37 | uncategorized | hypothetical protein MHA_0573 |
| Phl213 | MHA_0554 | 599205 | 599050 | 0.37 | uncategorized | hypothetical protein MHA_0574 |
| Phl213 | MHA_0555 | 599600 | 599343 | 0.37 | uncategorized | hypothetical protein MHA_0575 |
| Phl213 | MHA_0556 | 599614 | 599790 | 0.45 | uncategorized | hypothetical protein MHA_0576 |
| Phl213 | MHA_0557 | 599794 | 599952 | 0.43 | uncategorized | hypothetical protein MHA_0577 |
| Phl213 | MHA_0558 | 601123 | 600035 | 0.35 | COG1192 | possible chromosome partitioning ATPase Soj |
| Phl213 | MHA_0559 | 601619 | 601188 | 0.37 | uncategorized | hypothetical protein MHA_0579 |
| Phl213 | MHA_0577 | 618464 | 618814 | 0.34 | uncategorized | hypothetical protein MHA_0599 |
| Phl213 | MHA_0584 | 624483 | 626651 | 0.27 | COG0338 | site-specific DNA-methyltransferase (adenine-specific) |
| Phl213 | MHA_0585 | 626652 | 628357 | 0.31 | uncategorized | hypothetical protein MHA_0610 |
| Phl213 | MHA_0586 | 629540 | 628989 | 0.32 | uncategorized | hypothetical protein MHA_0611 |
| Phl213 | MHA_0587 | 629677 | 629501 | 0.36 | uncategorized | hypothetical protein MHA_0612 |
| Phl213 | MHA_0588 | 630164 | 629877 | 0.39 | uncategorized | hypothetical protein MHA_0613 |
| Phl213 | MHA_0589 | 630370 | 630624 | 0.43 | uncategorized | hypothetical protein MHA_0614 |
| Phl213 | MHA_0590 | 630628 | 630774 | 0.37 | uncategorized | hypothetical protein MHA_0615 |
| Phl213 | MHA_0591 | 630790 | 630996 | 0.37 | uncategorized | hypothetical protein MHA_0616 |
| Phl213 | MHA_0595 | 633606 | 633803 | 0.38 | uncategorized | hypothetical protein MHA_0621 |
| Phl213 | MHA_0598 | 634539 | 634282 | 0.37 | uncategorized | hypothetical protein MHA_0624 |
| Phl213 | MHA_0599 | 635051 | 634551 | 0.26 | uncategorized | hypothetical protein MHA_0625 |
| Phl213 | MHA_0603 | 638687 | 638785 | 0.23 | uncategorized | hypothetical protein MHA_0629 |
| Phl213 | MHA_0604 | 639060 | 639998 | 0.28 | COG0476 | possible molybdopterin/thiamine biosynthesis protein |
| Phl213 | MHA_0605 | 639995 | 641320 | 0.23 | uncategorized | hypothetical protein MHA_0631 |
| Phl213 | MHA_0606 | 641329 | 642606 | 0.26 | COG3842 | possible ABC superfamily ATP binding cassette transporter, ABC protein |
| Phl213 | MHA_0607 | 642593 | 643744 | 0.26 | COG0477 | possible MFS family major facilitator transporter |
| Phl213 | MHA_0608 | 643473 | 643279 | 0.28 | COG0477 | hypothetical protein MHA_0634 |
| Phl213 | MHA_0693 | 721884 | 723194 | 0.29 | uncategorized | hypothetical protein MHA_0724 |
| Phl213 | MHA_0765 | 792012 | 791182 | 0.43 | COG0627 | possible esterase |
| Phl213 | MHA_0864 | 913834 | 914496 | 0.38 | uncategorized | hypothetical protein MHA_0898 |
| Phl213 | MHA_0865 | 914489 | 916414 | 0.41 | COG2801 | possible recombinase |
| Phl213 | MHA_0881 | 924691 | 925038 | 0.39 | uncategorized | hypothetical protein MHA_0915 |
| Phl213 | MHA_0882 | 925164 | 925556 | 0.37 | uncategorized | hypothetical protein MHA_0916 |
| Phl213 | MHA_0912 | 946640 | 946957 | 0.43 | uncategorized | possible bacteriophage tail fiber protein |
| Phl213 | MHA_0913 | 947065 | 947307 | 0.31 | uncategorized | hypothetical protein MHA_0947 |
| Phl213 | MHA_0914 | 947308 | 947550 | 0.47 | uncategorized | hypothetical protein MHA_0948 |
| Phl213 | MHA_0916 | 947884 | 948450 | 0.41 | uncategorized | hypothetical protein MHA_0950 |
| Phl213 | MHA_0917 | 948408 | 949514 | 0.39 | uncategorized | hypothetical protein MHA_0951 |
| Phl213 | MHA_0918 | 949511 | 950863 | 0.40 | uncategorized | hypothetical protein MHA_0952 |
| Phl213 | MHA_0919 | 952695 | 951933 | 0.38 | uncategorized | hypothetical protein MHA_0953 |
| Phl213 | MHA_0920 | 953581 | 952688 | 0.40 | COG1961 | possible partitioning protein ParB |
| Phl213 | MHA_0921 | 955127 | 953496 | 0.40 | COG1961 | site-specific recombinase |
| Phl213 | MHA_0922 | 955175 | 955501 | 0.39 | uncategorized | hypothetical protein MHA_0956 |
| Phl213 | MHA_0923 | 955452 | 955661 | 0.34 | uncategorized | hypothetical protein MHA_0957 |
| Phl213 | MHA_0924 | 955887 | 956600 | 0.39 | uncategorized | hypothetical protein MHA_0958 |
| Phl213 | MHA_0925 | 956814 | 958433 | 0.29 | COG0286 | possible type IV site-specific deoxyribonuclease, methyltransferase subunit |
| Phl213 | MHA_0926 | 961480 | 958478 | 0.28 | COG0286 | possible type IV site-specific deoxyribonuclease, methyltransferase and restriction subunits |
| Phl213 | MHA_0927 | 964282 | 961502 | 0.37 | COG0210 | possible helicase |
| Phl213 | MHA_1037 | 1089734 | 1089579 | 0.34 | uncategorized | hypothetical protein MHA_1081 |
| Phl213 | MHA_1038 | 1090057 | 1089680 | 0.36 | uncategorized | hypothetical protein MHA_1082 |
| Phl213 | MHA_1046 | 1092720 | 1093013 | 0.38 | uncategorized | hypothetical protein MHA_1090 |
| Phl213 | MHA_1047 | 1093016 | 1093225 | 0.37 | uncategorized | hypothetical bacteriophage protein |
| Phl213 | MHA_1048 | 1093372 | 1093497 | 0.28 | uncategorized | hypothetical protein MHA_1092 |
| Phl213 | MHA_1049 | 1093544 | 1094386 | 0.33 | uncategorized | hypothetical protein MHA_1093 |
| Phl213 | MHA_1102 | 1141725 | 1141327 | 0.32 | COG0789 | possible MerR family transcriptional regulator |
| Phl213 | MHA_1103 | 1141797 | 1142411 | 0.41 | COG0053 | CDF family cation diffusion facilitator |
| Phl213 | MHA_1104 | 1143682 | 1143293 | 0.46 | COG0789 | possible MerR family transcriptional regulator |
| Phl213 | MHA_1105 | 1143682 | 1143837 | 0.33 | uncategorized | hypothetical protein MHA_1152 |
| Phl213 | MHA_1106 | 1144119 | 1143877 | 0.49 | COG3544 | hypothetical protein MHA_1153 |
| Phl213 | MHA_1107 | 1144090 | 1144833 | 0.48 | COG3544 | hypothetical protein MHA_1154 |
| Phl213 | MHA_1108 | 1145052 | 1145423 | 0.32 | COG3759 | possible membrane protein |
| Phl213 | MHA_1109 | 1145446 | 1145895 | 0.38 | COG1846 | possible MarR family transcriptional regulator |
| Phl213 | MHA_1110 | 1145934 | 1146266 | 0.46 | COG2076 | DMT superfamily drug/metabolite transporter |
| Phl213 | MHA_1111 | 1147569 | 1146367 | 0.41 | COG0477 | MFS family major facilitator tetracycline transporter |
| Phl213 | MHA_1112 | 1147660 | 1148283 | 0.40 | COG1309 | tetracycline resistance repressor TetR |
| Phl213 | MHA_1113 | 1148804 | 1148286 | 0.42 | uncategorized | hypothetical protein MHA_1160 |
| Phl213 | MHA_1114 | 1149032 | 1148853 | 0.41 | uncategorized | hypothetical protein MHA_1161 |
| Phl213 | MHA_1115 | 1150913 | 1149366 | 0.42 | COG2132 | multicopper (Cu2+) oxidase |
| Phl213 | MHA_1116 | 1151381 | 1150926 | 0.41 | COG3019 | possible metal-binding protein |
| Phl213 | MHA_1117 | 1151883 | 1151563 | 0.36 | uncategorized | hypothetical protein MHA_1166 |
| Phl213 | MHA_1118 | 1151882 | 1152649 | 0.44 | COG0667 | possible dehydrogenase |
| Phl213 | MHA_1119 | 1153898 | 1153188 | 0.31 | uncategorized | hypothetical protein MHA_1168 |
| Phl213 | MHA_1120 | 1153994 | 1154278 | 0.40 | uncategorized | hypothetical protein MHA_1169 |
| Phl213 | MHA_1124 | 1158462 | 1159142 | 0.38 | uncategorized | hypothetical protein MHA_1173 |
| Phl213 | MHA_1151 | 1179010 | 1178687 | 0.30 | uncategorized | hypothetical protein MHA_1201 |
| Phl213 | MHA_1152 | 1179517 | 1179023 | 0.35 | COG0454 | hypothetical protein MHA_1202 |
| Phl213 | MHA_1153 | 1179789 | 1179517 | 0.41 | COG4453 | hypothetical membrane protein |
| Phl213 | MHA_1154 | 1179771 | 1179872 | 0.31 | uncategorized | hypothetical protein MHA_1204 |
| Phl213 | MHA_1182 | 1210541 | 1210936 | 0.40 | uncategorized | possible bacteriophage tail protein |
| Phl213 | MHA_1183 | 1210947 | 1212815 | 0.43 | COG3378 | bacteriophage protein |
| Phl213 | MHA_1184 | 1214215 | 1212974 | 0.37 | COG0582 | bacteriophage integrase |
| Phl213 | MHA_1221 | 1245920 | 1246408 | 0.46 | uncategorized | hypothetical bacteriophage protein |
| Phl213 | MHA_1222 | 1247144 | 1247386 | 0.47 | uncategorized | hypothetical protein MHA_1274 |
| Phl213 | MHA_1422 | 1452565 | 1453155 | 0.38 | uncategorized | hypothetical protein MHA_1477 |
| Phl213 | MHA_1423 | 1453225 | 1453743 | 0.41 | uncategorized | hypothetical protein MHA_1478 |
| Phl213 | MHA_1424 | 1454201 | 1453890 | 0.38 | uncategorized | hypothetical protein MHA_1479 |
| Phl213 | MHA_1425 | 1454343 | 1454194 | 0.37 | uncategorized | hypothetical protein MHA_1480 |
| Phl213 | MHA_1429 | 1464061 | 1463894 | 0.35 | uncategorized | hypothetical protein MHA_1484 |
| Phl213 | MHA_1432 | 1465940 | 1465611 | 0.41 | COG4718 | possible bacteriophage tail protein |
| Phl213 | MHA_1433 | 1469506 | 1465940 | 0.43 | COG5281 | possible bacteriophage tail protein |
| Phl213 | MHA_1434 | 1469787 | 1469560 | 0.35 | uncategorized | hypothetical protein MHA_1489 |
| Phl213 | MHA_1435 | 1469881 | 1469780 | 0.37 | uncategorized | hypothetical protein MHA_1490 |
| Phl213 | MHA_1436 | 1470080 | 1469850 | 0.48 | uncategorized | hypothetical protein MHA_1491 |
| Phl213 | MHA_1437 | 1470526 | 1470125 | 0.43 | uncategorized | hypothetical protein MHA_1492 |
| Phl213 | MHA_1439 | 1471674 | 1471279 | 0.38 | uncategorized | possible bacteriophage tail protein |
| Phl213 | MHA_1440 | 1472195 | 1471671 | 0.47 | uncategorized | possible bacteriophage tail protein |
| Phl213 | MHA_1441 | 1472501 | 1472199 | 0.48 | uncategorized | hypothetical protein MHA_1496 |
| Phl213 | MHA_1442 | 1472817 | 1472494 | 0.44 | uncategorized | hypothetical protein MHA_1497 |
| Phl213 | MHA_1443 | 1474852 | 1472891 | 0.47 | COG0740 | S14 family bacteriophage protease |
| Phl213 | MHA_1444 | 1476363 | 1474864 | 0.50 | COG5511 | bacteriophage capsid protein |
| Phl213 | MHA_1445 | 1476587 | 1476363 | 0.44 | uncategorized | hypothetical protein MHA_1500 |
| Phl213 | MHA_1446 | 1478695 | 1476584 | 0.50 | COG5525 | bacteriophage terminase large subunit |
| Phl213 | MHA_1447 | 1479171 | 1478695 | 0.45 | uncategorized | hypothetical bacteriophage protein |
| Phl213 | MHA_1683 | 1703703 | 1702912 | 0.39 | uncategorized | possible bacteriophage replication protein |
| Phl213 | MHA_1685 | 1704734 | 1704510 | 0.41 | uncategorized | hypothetical bacteriophage protein |
| Phl213 | MHA_1687 | 1705597 | 1706100 | 0.34 | uncategorized | hypothetical protein MHA_1754 |
| Phl213 | MHA_1688 | 1706097 | 1706896 | 0.34 | uncategorized | hypothetical protein MHA_1755 |
| Phl213 | MHA_1776 | 1795056 | 1794475 | 0.30 | uncategorized | hypothetical protein MHA_1847 |
| Phl213 | MHA_1778 | 1798098 | 1797163 | 0.28 | COG0463 | possible glycosyltransferase |
| Phl213 | MHA_1779 | 1799295 | 1798126 | 0.25 | uncategorized | hypothetical protein MHA_1851 |
| Phl213 | MHA_1780 | 1800265 | 1799318 | 0.25 | uncategorized | possible sialyltransferase |
| Phl213 | MHA_1842 | 1856113 | 1855511 | 0.31 | uncategorized | hypothetical protein MHA_1915 |
| Phl213 | MHA_1899 | 1899849 | 1899145 | 0.45 | uncategorized | hypothetical protein MHA_1968 |
| Phl213 | MHA_1900 | 1900006 | 1899842 | 0.42 | uncategorized | hypothetical protein MHA_1969 |
| Phl213 | MHA_1901 | 1900303 | 1900016 | 0.44 | uncategorized | hypothetical protein MHA_1970 |
| Phl213 | MHA_1905 | 1904020 | 1903454 | 0.44 | COG4385 | possible bacteriophage tail protein |
| Phl213 | MHA_1906 | 1905116 | 1904013 | 0.43 | COG3299 | possible bacteriophage baseplate protein |
| Phl213 | MHA_1907 | 1905488 | 1905126 | 0.42 | COG3628 | possible bacteriophage baseplate protein |
| Phl213 | MHA_1908 | 1906079 | 1905543 | 0.50 | COG4540 | possible bacteriophage baseplate protein |
| Phl213 | MHA_1909 | 1907130 | 1906066 | 0.44 | COG3500 | possible bacteriophage protein |
| Phl213 | MHA_1910 | 1907350 | 1907123 | 0.44 | uncategorized | possible bacteriophage tail fiber protein |
| Phl213 | MHA_1911 | 1908257 | 1907334 | 0.48 | uncategorized | hypothetical bacteriophage protein |
| Phl213 | MHA_1913 | 1910972 | 1911274 | 0.43 | uncategorized | hypothetical protein MHA_1982 |
| Phl213 | MHA_1914 | 1911716 | 1911402 | 0.39 | uncategorized | hypothetical protein MHA_1983 |
| Phl213 | MHA_1915 | 1912327 | 1911812 | 0.44 | COG3498 | possible bacteriophage tail core protein |
| Phl213 | MHA_1916 | 1913723 | 1912338 | 0.49 | COG3497 | bacteriophage tail sheath protein |
| Phl213 | MHA_1917 | 1914305 | 1913808 | 0.48 | uncategorized | hypothetical bacteriophage protein |
| Phl213 | MHA_1918 | 1914745 | 1914311 | 0.47 | COG4387 | hypothetical bacteriophage protein |
| Phl213 | MHA_1919 | 1915026 | 1914745 | 0.43 | uncategorized | hypothetical protein MHA_1988 |
| Phl213 | MHA_1920 | 1916021 | 1915095 | 0.46 | uncategorized | hypothetical bacteriophage protein |
| Phl213 | MHA_1921 | 1917167 | 1916052 | 0.47 | uncategorized | hypothetical bacteriophage protein |
| Phl213 | MHA_1922 | 1917866 | 1917402 | 0.46 | COG5005 | hypothetical bacteriophage protein |
| Phl213 | MHA_1923 | 1917930 | 1918274 | 0.37 | uncategorized | hypothetical protein MHA_1992 |
| Phl213 | MHA_1924 | 1918404 | 1918225 | 0.33 | uncategorized | hypothetical protein MHA_1993 |
| Phl213 | MHA_1927 | 1921315 | 1921184 | 0.36 | uncategorized | hypothetical protein MHA_1996 |
| Phl213 | MHA_1932 | 1924255 | 1924103 | 0.42 | uncategorized | hypothetical protein MHA_2001 |
| Phl213 | MHA_1934 | 1924892 | 1924623 | 0.38 | uncategorized | hypothetical protein MHA_2003 |
| Phl213 | MHA_1936 | 1925899 | 1925540 | 0.39 | COG5566 | possible bacteriophage transcriptional regulator |
| Phl213 | MHA_1939 | 1927535 | 1927248 | 0.36 | uncategorized | hypothetical protein MHA_2008 |
| Phl213 | MHA_1943 | 1928815 | 1928522 | 0.40 | uncategorized | hypothetical protein MHA_2012 |
| Phl213 | MHA_1949 | 1933176 | 1932985 | 0.40 | uncategorized | possible bacteriophage transcriptional regulator |
| Phl213 | MHA_1950 | 1933417 | 1934094 | 0.43 | COG1974 | possible bacteriophage transcriptional regulator |
| Phl213 | MHA_2042 | 2029232 | 2029438 | 0.29 | uncategorized | hypothetical protein MHA_2116 |
| Phl213 | MHA_2043 | 2029359 | 2029583 | 0.40 | uncategorized | hypothetical protein MHA_2117 |
| Phl213 | MHA_2044 | 2029626 | 2029955 | 0.34 | COG4226 | possible pilus related protein HicB |
| Phl213 | MHA_2257 | 2238144 | 2238827 | 0.37 | COG0500 | hypothetical protein MHA_2333 |
| Phl213 | MHA_2265 | 2243140 | 2242655 | 0.43 | uncategorized | cytochrome c biogenesis protein |
| Phl213 | MHA_2266 | 2244128 | 2243091 | 0.23 | uncategorized | hypothetical protein MHA_2342 |
| Phl213 | MHA_2354 | 2332674 | 2332778 | 0.34 | COG1974 | hypothetical protein MHA_2435 |
| Phl213 | MHA_2355 | 2333400 | 2332741 | 0.42 | COG1974 | possible LexA family repressor/S24 family protease |
| Phl213 | MHA_2356 | 2333530 | 2333736 | 0.40 | uncategorized | possible Cro repressor |
| Phl213 | MHA_2359 | 2334181 | 2334351 | 0.36 | uncategorized | hypothetical protein MHA_2440 |
| Phl213 | MHA_2360 | 2334326 | 2335195 | 0.39 | uncategorized | possible bacteriophage replication protein |
| Phl213 | MHA_2383 | 2343841 | 2345244 | 0.40 | COG5585 | hypothetical bacteriophage protein |
| Phl213 | MHA_2384 | 2345192 | 2346853 | 0.40 | COG5585 | hypothetical bacteriophage protein |
| Phl213 | MHA_2385 | 2346853 | 2347071 | 0.38 | uncategorized | hypothetical bacteriophage protein |
| Phl213 | MHA_2386 | 2347072 | 2347485 | 0.38 | COG0317 | hypothetical bacteriophage protein |
| Phl213 | MHA_2388 | 2348403 | 2349350 | 0.46 | COG5492 | hypothetical bacteriophage protein |
| Phl213 | MHA_2389 | 2349410 | 2349760 | 0.39 | uncategorized | hypothetical protein MHA_2469 |
| Phl213 | MHA_2390 | 2349750 | 2350187 | 0.44 | uncategorized | hypothetical bacteriophage protein |
| Phl213 | MHA_2391 | 2350187 | 2350558 | 0.45 | uncategorized | hypothetical protein MHA_2471 |
| Phl213 | MHA_2392 | 2350551 | 2350964 | 0.46 | uncategorized | hypothetical bacteriophage protein |
| Phl213 | MHA_2393 | 2350964 | 2351356 | 0.49 | uncategorized | hypothetical protein MHA_2473 |
| Phl213 | MHA_2394 | 2351366 | 2352388 | 0.48 | uncategorized | hypothetical protein MHA_2474 |
| Phl213 | MHA_2395 | 2352478 | 2352879 | 0.46 | uncategorized | hypothetical protein MHA_2475 |
| Phl213 | MHA_2396 | 2352894 | 2353220 | 0.45 | uncategorized | hypothetical protein MHA_2476 |
| Phl213 | MHA_2397 | 2353222 | 2353461 | 0.46 | uncategorized | bacteriophage tail protein |
| Phl213 | MHA_2438 | 2391024 | 2390548 | 0.30 | uncategorized | hypothetical protein MHA_2512 |
| Phl213 | MHA_2439 | 2391753 | 2391169 | 0.24 | uncategorized | hypothetical protein MHA_2513 |
| Phl213 | MHA_2440 | 2392888 | 2391755 | 0.35 | COG1450 | PulD family bacteriophage secretion protein |
| Phl213 | MHA_2441 | 2394083 | 2393928 | 0.30 | uncategorized | hypothetical bacteriophage protein |
| Phl213 | MHA_2508 | 2456249 | 2456395 | 0.40 | uncategorized | hypothetical protein MHA_2585 |
| Phl213 | MHA_2509 | 2456292 | 2457500 | 0.45 | uncategorized | Tn10 family transposase |
| Phl213 | MHA_2513 | 2459428 | 2459535 | 0.35 | uncategorized | hypothetical protein MHA_2590 |
| Phl213 | MHA_2514 | 2459732 | 2459938 | 0.41 | uncategorized | possible bacteriophage transcriptional regulator |
| Phl213 | MHA_2515 | 2459948 | 2460607 | 0.31 | uncategorized | hypothetical protein MHA_2592 |
| Phl213 | MHA_2516 | 2460618 | 2461568 | 0.42 | uncategorized | possible bacteriophage major capsid protein |
| Phl213 | MHA_2517 | 2461565 | 2461759 | 0.42 | uncategorized | hypothetical protein MHA_2594 |
| Phl213 | MHA_2518 | 2461877 | 2462725 | 0.30 | uncategorized | hypothetical protein MHA_2595 |
| Phl213 | MHA_2519 | 2462803 | 2463054 | 0.40 | uncategorized | possible bacteriophage transcriptional regulator |
| Phl213 | MHA_2520 | 2463054 | 2463434 | 0.45 | uncategorized | hypothetical protein MHA_2597 |
| Phl213 | MHA_2521 | 2463502 | 2463684 | 0.31 | uncategorized | hypothetical protein MHA_2598 |
| Phl213 | MHA_2526 | 2466730 | 2466629 | 0.33 | uncategorized | hypothetical protein MHA_2604 |
| Phl213 | MHA_2527 | 2468574 | 2468864 | 0.30 | uncategorized | hypothetical protein MHA_2606 |
| Phl213 | MHA_2528 | 2469647 | 2469543 | 0.34 | uncategorized | hypothetical protein MHA_2607 |
| Phl213 | MHA_2529 | 2469851 | 2470174 | 0.45 | COG3636 | possible bacteriophage transcriptional regulator |
| Phl213 | MHA_2538 | 2473862 | 2474098 | 0.35 | uncategorized | hypothetical protein MHA_2619 |
| Phl213 | MHA_2545 | 2477362 | 2478156 | 0.44 | uncategorized | bacteriophage recombinase |
| Phl213 | MHA_2546 | 2478153 | 2478788 | 0.46 | uncategorized | possible bacteriophage exonuclease |
| Phl213 | MHA_2572 | 2496397 | 2496720 | 0.44 | COG3636 | possible transcriptional regulator |
| Phl213 | MHA_2575 | 2497811 | 2497933 | 0.28 | uncategorized | hypothetical protein MHA_2658 |
| Phl213 | MHA_2642 | 2561756 | 2561857 | 0.33 | uncategorized | hypothetical protein MHA_2725 |
| Phl213 | MHA_2733 | 2651109 | 2650000 | 0.26 | uncategorized | possible membrane protein |
| Phl213 | MHA_2734 | 2651305 | 2651081 | 0.30 | uncategorized | hypothetical protein MHA_2821 |
| Phl213 | MHA_2735 | 2651997 | 2651455 | 0.31 | uncategorized | hypothetical protein MHA_2822 |
