# Supplementary material for: A three-way comparative genomic analysis of Mannheimia haemolytica isolates
Source: BMC Genomics. 2010 Oct 4;11:535. doi: 10.1186/1471-2164-11-535 (PMC3091684; doi:10.1186/1471-2164-11-535)
Supplement: Additional file 2 — Table S2: M. haemolytica Bovine (B) specific genes. [file 1471-2164-11-535-S2.DOC]

**Table S2:** *M. haemolytica* Bovine (B) specific genes

| **Contig ID** | **Gene ID** | **Start** | **Stop** | **%GC** | **COG** | **Product** |
| --- | --- | --- | --- | --- | --- | --- |
| contig00003 | COK_0080 | 76879 | 77457 | 0.29 | uncategorized | hypothetical protein |
| contig00015 | COK_0537 | 1274 | 486 | 0.45 | uncategorized | hypothetical protein |
| contig00015 | COK_0538 | 2641 | 1274 | 0.45 | COG4228 | Phage tail/DNA circulation protein |
| contig00015 | COK_0539 | 4558 | 2651 | 0.47 | COG3941 | hypothetical protein |
| contig00015 | COK_0540 | 5031 | 4648 | 0.48 | COG4518 | hypothetical protein |
| contig00015 | COK_0541 | 5387 | 5034 | 0.45 | uncategorized | hypothetical protein |
| contig00015 | COK_0542 | 6861 | 5398 | 0.45 | COG4386 | Bacteriophage tail sheath protein |
| contig00015 | COK_0543 | 7049 | 6861 | 0.39 | uncategorized | hypothetical protein |
| contig00015 | COK_0544 | 7615 | 7049 | 0.46 | COG5003 | hypothetical protein |
| contig00015 | COK_0545 | 8031 | 7612 | 0.47 | COG4387 | Mu-like prophage protein GP36 |
| contig00015 | COK_0546 | 8417 | 8031 | 0.46 | uncategorized | hypothetical protein |
| contig00015 | COK_0547 | 9419 | 8496 | 0.45 | COG4397 | Phage major capsid protein |
| contig00015 | COK_0548 | 10474 | 9419 | 0.48 | COG4388 | hypothetical protein |
| contig00022 | COK_0658 | 8284 | 7253 | 0.30 | uncategorized | Putative deoxyribonuclease YjjV |
| contig00023 | COK_0722 | 26776 | 26660 | 0.36 | uncategorized | hypothetical protein |
| contig00023 | COK_0727 | 28926 | 28243 | 0.43 | uncategorized | hypothetical protein |
| contig00033 | COK_1021 | 87160 | 87432 | 0.44 | uncategorized | hypothetical protein |
| contig00033 | COK_1024 | 89076 | 89744 | 0.32 | uncategorized | hypothetical protein |
| contig00033 | COK_1025 | 89797 | 90240 | 0.36 | uncategorized | Exonuclease SbcC |
| contig00033 | COK_1028 | 92305 | 92141 | 0.32 | uncategorized | hypothetical protein |
| contig00039 | COK_1259 | 37477 | 38676 | 0.40 | COG0582 | phage integrase family protein |
| contig00039 | COK_1260 | 38798 | 38673 | 0.46 | uncategorized | hypothetical protein |
| contig00039 | COK_1263 | 39451 | 40128 | 0.40 | uncategorized | hypothetical protein |
| contig00039 | COK_1264 | 40149 | 40871 | 0.42 | uncategorized | Phage capsid scaffolding protein |
| contig00039 | COK_1265 | 40886 | 41905 | 0.42 | uncategorized | Phage capsid protein |
| contig00039 | COK_1266 | 41916 | 42560 | 0.40 | uncategorized | Putative deoxyribonuclease YcfH |
| contig00039 | COK_1267 | 42667 | 43629 | 0.34 | uncategorized | hypothetical protein |
| contig00039 | COK_1268 | 43781 | 43626 | 0.37 | uncategorized | hypothetical protein |
| contig00039 | COK_1269 | 43804 | 44004 | 0.43 | uncategorized | hypothetical protein |
| contig00039 | COK_1274 | 45759 | 46061 | 0.38 | uncategorized | hypothetical protein |
| contig00039 | COK_1275 | 46110 | 47051 | 0.28 | uncategorized | Zinc binding domain / DNA primase2C Phage P4-associated / Replicative helicase RepA2C Phage P4-associated |
| contig00039 | COK_1276 | 47032 | 47502 | 0.27 | uncategorized | bacteriophage protein |
| contig00229 | COK_1592 | 2366 | 3013 | 0.44 | uncategorized | Phage DNA binding ATPase |
| contig00240 | COK_1887 | 27231 | 27064 | 0.41 | uncategorized | Valyl-tRNA synthetase |
| contig00246 | COK_2140 | 2033 | 1902 | 0.22 | uncategorized | hypothetical protein |
| contig00251 | COK_2259 | 16588 | 15236 | 0.40 | COG2211 | Melibiose carrier protein2C Na+/melibiose symporter |
| contig00251 | COK_2260 | 18424 | 16664 | 0.38 | uncategorized | Beta-glucosidase-related glycosidases |
| contig00261 | COK_2410 | 6999 | 6121 | 0.30 | COG2990 | Membrane protein LAPB |
