# Supplementary material for: A three-way comparative genomic analysis of Mannheimia haemolytica isolates
Source: BMC Genomics. 2010 Oct 4;11:535. doi: 10.1186/1471-2164-11-535 (PMC3091684; doi:10.1186/1471-2164-11-535)
Supplement: Additional file 3 — Table S3: M. haemolytica Ovine (O) specific genes. [file 1471-2164-11-535-S3.DOC]

**Table S3:** *M. haemolytica* Ovine (O) specific genes

| **Contig ID** | **Gene ID** | **Start** | **Stop** | **%GC** | **COG** | **Product** |
| --- | --- | --- | --- | --- | --- | --- |
| contig00004 | COI_0077 | 1531 | 1977 | 0.29 | uncategorized | hypothetical protein |
| contig00004 | COI_0078 | 1978 | 2577 | 0.32 | uncategorized | hypothetical protein |
| contig00013 | COI_0195 | 657 | 130 | 0.26 | uncategorized | hypothetical protein |
| contig00022 | COI_0458 | 27186 | 25714 | 0.42 | COG2610 | H+/gluconate symporter and related permeases |
| contig00022 | COI_0459 | 28148 | 27201 | 0.40 | COG0451 | Nucleoside-diphosphate-sugar epimerases |
| contig00028 | COI_0675 | 1895 | 2794 | 0.44 | uncategorized | Putative bacteriophage protein |
| contig00038 | COI_0907 | 17139 | 16141 | 0.40 | COG1638 | TRAP-type C4-dicarboxylate transport system2C periplasmic component |
| contig00038 | COI_0908 | 17290 | 17856 | 0.37 | COG3090 | TRAP-type transport system2C small permease component2C predicted N-acetylneuraminate transporter |
| contig00038 | COI_0909 | 17873 | 19174 | 0.41 | COG1593 | TRAP-type C4-dicarboxylate transport system2C large permease component |
| contig00043 | COI_1006 | 9516 | 8773 | 0.42 | uncategorized | hypothetical protein |
| contig00043 | COI_1007 | 10466 | 9747 | 0.46 | COG0846 | NAD-dependent protein deacetylase of SIR2 family |
| contig00043 | COI_1008 | 11115 | 10564 | 0.38 | uncategorized | hypothetical protein |
| contig00043 | COI_1009 | 11394 | 11693 | 0.39 | uncategorized | hypothetical protein |
| contig00043 | COI_1010 | 11690 | 11839 | 0.39 | uncategorized | hypothetical protein |
| contig00043 | COI_1011 | 12293 | 11853 | 0.44 | uncategorized | hypothetical protein |
| contig00043 | COI_1012 | 13330 | 12293 | 0.44 | uncategorized | hypothetical protein |
| contig00043 | COI_1013 | 13535 | 13777 | 0.36 | uncategorized | hypothetical protein |
| contig00043 | COI_1014 | 14672 | 13821 | 0.31 | uncategorized | hypothetical protein |
| contig00051 | COI_1025 | 7231 | 6902 | 0.42 | COG4718 | minor tail protein |
| contig00051 | COI_1026 | 9730 | 7235 | 0.43 | uncategorized | Phage tail length tape-measure protein 1 |
| contig00051 | COI_1028 | 10738 | 10280 | 0.33 | uncategorized | diguanylate cyclase/phosphodiesterase (GGDEF & EAL domains) with PAS/PAC sensor(s) |
| contig00051 | COI_1029 | 10871 | 11140 | 0.36 | uncategorized | hypothetical protein |
| contig00051 | COI_1030 | 11826 | 11155 | 0.40 | uncategorized | hypothetical protein |
| contig00051 | COI_1031 | 11922 | 12098 | 0.43 | uncategorized | hypothetical protein |
| contig00051 | COI_1032 | 12154 | 12570 | 0.44 | COG1598 | hypothetical protein |
| contig00051 | COI_1033 | 13092 | 12610 | 0.45 | uncategorized | hypothetical protein |
| contig00051 | COI_1034 | 13476 | 13096 | 0.43 | uncategorized | hypothetical protein |
| contig00051 | COI_1035 | 13841 | 13473 | 0.46 | uncategorized | hypothetical protein |
| contig00051 | COI_1036 | 14187 | 13843 | 0.48 | uncategorized | hypothetical protein |
| contig00051 | COI_1037 | 14564 | 14187 | 0.49 | uncategorized | hypothetical protein |
| contig00051 | COI_1038 | 14731 | 14567 | 0.44 | uncategorized | hypothetical protein |
| contig00051 | COI_1039 | 15765 | 14776 | 0.46 | uncategorized | hypothetical protein |
| contig00051 | COI_1040 | 16214 | 15780 | 0.50 | uncategorized | hypothetical protein |
| contig00051 | COI_1041 | 17562 | 16207 | 0.46 | uncategorized | hypothetical protein |
| contig00051 | COI_1042 | 18439 | 17564 | 0.46 | uncategorized | COG0840: Methyl-accepting chemotaxis protein |
| contig00051 | COI_1043 | 19832 | 18456 | 0.46 | uncategorized | COG3567: Uncharacterized protein conserved in bacteria |
| contig00064 | COI_1098 | 20185 | 20562 | 0.41 | uncategorized | hypothetical protein |
| contig00064 | COI_1124 | 35894 | 36160 | 0.37 | uncategorized | hypothetical protein |
| contig00066 | COI_1137 | 565 | 428 | 0.40 | uncategorized | hypothetical protein |
| contig00066 | COI_1138 | 958 | 839 | 0.36 | uncategorized | hypothetical protein |
| contig00069 | COI_1238 | 1805 | 1356 | 0.36 | uncategorized | hypothetical protein |
| contig00073 | COI_1464 | 589 | 957 | 0.23 | uncategorized | C-5 cytosine-specific DNA methylase |
| contig00077 | COI_1520 | 440 | 168 | 0.32 | uncategorized | hypothetical protein |
| contig00079 | COI_1524 | 614 | 1537 | 0.43 | uncategorized | Recombinational DNA repair protein RecT (prophage associated) |
| contig00079 | COI_1525 | 1515 | 2192 | 0.45 | uncategorized | hypothetical protein |
| contig00081 | COI_1539 | 39 | 293 | 0.29 | uncategorized | Cell division protein FtsX |
| contig00107 | COI_1701 | 140 | 397 | 0.36 | uncategorized | hypothetical protein |
| contig00111 | COI_1732 | 664 | 783 | 0.38 | uncategorized | hypothetical protein |
| contig00130 | COI_2062 | 737 | 525 | 0.40 | uncategorized | hypothetical protein |
| contig00134 | COI_2064 | 160 | 20 | 0.35 | uncategorized | COG0270: Site-specific DNA methylase |
| contig00148 | COI_2158 | 17499 | 18161 | 0.34 | uncategorized | hypothetical protein |
| contig00148 | COI_2159 | 18184 | 18855 | 0.31 | uncategorized | hypothetical protein |
| contig00150 | COI_2171 | 1126 | 947 | 0.45 | uncategorized | hypothetical protein |
| contig00150 | COI_2178 | 4903 | 4679 | 0.33 | uncategorized | hypothetical protein |
| contig00156 | COI_2421 | 91 | 324 | 0.39 | uncategorized | predicted Endodeoxyribonuclease RusA |
| contig00156 | COI_2424 | 1095 | 922 | 0.37 | uncategorized | hypothetical protein |
| contig00171 | COI_2711 | 104 | 1081 | 0.34 | uncategorized | Replication protein |
| contig00171 | COI_2712 | 1091 | 1297 | 0.33 | uncategorized | Haemophilus-specific protein2C uncharacterized |
| contig00171 | COI_2713 | 1305 | 1502 | 0.36 | uncategorized | hypothetical protein |
| contig00171 | COI_2714 | 1513 | 1731 | 0.32 | uncategorized | hypothetical protein |
| contig00171 | COI_2715 | 1731 | 2243 | 0.31 | uncategorized | hypothetical protein |
| contig00179 | COI_2732 | 1497 | 1312 | 0.34 | uncategorized | hypothetical protein |
