# Supplementary material for: A three-way comparative genomic analysis of Mannheimia haemolytica isolates
Source: BMC Genomics. 2010 Oct 4;11:535. doi: 10.1186/1471-2164-11-535 (PMC3091684; doi:10.1186/1471-2164-11-535)
Supplement: Additional file 4 — Table S4: Summary of data used for precision analysis and precision statistics. [file 1471-2164-11-535-S4.DOC]

**Table S4:** Summary of data used for precision analysis and precision statistics

|  | **Set 1** | **Set 2** |
| --- | --- | --- |
| Total number of reads | 299,583 | 299,584 |
| Total number of bases | 116,080,367 bp | 116,155,583 bp |
| Total number of large contigs (>=500 bp) | 100 (7,182,188 bp) | 94 (7,181,898 bp) |
| Average contig size | 71,821 bp | 76,403 |
|  | **Set 1 mapped to Set 2** | **Set 2 mapped to Set 1** |
| Total aligned length | 6,506,615 bp | 6,128,403 bp |
| Total differences | 368 | 341 |
| Total rate of differences | 1/17681 bp | 1/17971 bp |
| Total non-homopolymer differences | 88 (24%) | 86 (25%) |
| Total homopolymer differences | 280 (76 %) | 255 (75%) |
