# Supplementary material for: A three-way comparative genomic analysis of Mannheimia haemolytica isolates
Source: BMC Genomics. 2010 Oct 4;11:535. doi: 10.1186/1471-2164-11-535 (PMC3091684; doi:10.1186/1471-2164-11-535)
Supplement: Additional file 5 — Figure S5: Multiple sequence alignment of LktA DNA and protein sequences. [file 1471-2164-11-535-S5.DOC]

**Figure S5:** Multiple sequence alignment of LktA DNA and protein sequences.

A1-MHA_0254 ATGGGAACTAGACTTACAACCCTATCAAATGGGCTAAAAAACACTTTAACGGCAACCAAA

B-COK_0274 ATGGGTAATAAATTTACTAATATTTCAACAAACCTAAGAAATTCGTGGCTTACAGCCAAA

O-COI_0481 ATGGGTAATAAATTTACTAATATTTCAACAAACCTAAGAAATTCGTGGCTTACAGCCAAA

DNA Homology ***** * ** * **** * * **** **** *** * * ** *****

A1-MHA_0254 M G T R L T T L S N G L K N T L T A T K

B-COK_0274 M G N K F T N I S T N L R N S W L T A K

O-COI_0481 M G N K F T N I S T N L R N S W L T A K

Protein Homology * * . : : * . : * . . * : * *

A1-MHA_0254 AGTGGCTTACATAAAGCCGGTCAATCATTAACCCAAGCCGGCAGTTCTTTAAAAACTGGG

B-COK_0274 TCTGGTTTAAATAACGCAGGACAATCATTAGCCAAAGCAGGGCAATCTTTAAAAACAGGG

O-COI_0481 TCTGGTTTAAATAACGCAGGACAATCATTAGCCAAAGCAGGGCAATCTTTAAAAACAGGG

DNA Homology *** *** **** ** ** ********* ** **** ** *********** ***

A1-MHA_0254 S G L H K A G Q S L T Q A G S S L K T G

B-COK_0274 S G L N N A G Q S L A K A G Q S L K T G

O-COI_0481 S G L N N A G Q S L A K A G Q S L K T G

Protein Homology * * * : : * * * * * : : * * . * * * * *

A1-MHA_0254 GCAAAAAAAATTATCCTCTATATTCCCCAAAATTACCAATATGATACTGAACAAGGTAAT

B-COK_0274 GCAAAAAAAATCATCCTCTATATCCCAAAAGATTACCAATATGATACCGATAAAGGTAAT

O-COI_0481 GCAAAAAAAATTATCCTCTATATTCCCCAAAATTACCAATATGATACTGAACAAGGTAAT

DNA Homology *********** *********** ** ** **************** ** ********

A1-MHA_0254 A K K I I L Y I P Q N Y Q Y D T E Q G N

B-COK_0274 A K K I I L Y I P K D Y Q Y D T D K G N

O-COI_0481 A K K I I L Y I P Q N Y Q Y D T E Q G N

Protein Homology * * * * * * * * * : : * * * * * : : * *

A1-MHA_0254 GGTTTACAGGATTTAGTCAAAGCGGCCGAAGAGTTGGGGATTGAGGTACAAAGAGAAGAA

B-COK_0274 GGTTTACAGGATTTAGTTAAGGCAGCTGAAGAGCTTGGTATTGAAGTGCAAAAAGAAGAG

O-COI_0481 GGTTTACAGGATTTAGTCAAAGCGGCCGAAGAGTTGGGGATTGAGGTACAAAGAGAAGAA

DNA Homology ***************** ** ** ** ****** * ** ***** ** **** ******

A1-MHA_0254 G L Q D L V K A A E E L G I E V Q R E E

B-COK_0274 G L Q D L V K A A E E L G I E V Q K E E

O-COI_0481 G L Q D L V K A A E E L G I E V Q R E E

Protein Homology * * * * * * * * * * * * * * * * * : * *

A1-MHA_0254 CGCAATAATATTGCAACAGCTCAAACCAGTTTAGGCACGATTCAAACCGCTATTGGCTTA

B-COK_0274 AGCAATGATATTGCAAAAGCACAAACCAGTTTAGGCACAATTCACAATGTATTGGGTTTA

O-COI_0481 CGCAATAATATTGCAACAGCTCAAACCAGTTTAGGCACGATTCAAACCGCTATTGGCTTA

DNA Homology ***** ********* *** ***************** ***** * * * ** ***

A1-MHA_0254 R N N I A T A Q T S L G T I Q T A I G L

B-COK_0274 S N D I A K A Q T S L G T I H N V L G L

O-COI_0481 R N N I A T A Q T S L G T I Q T A I G L

Protein Homology * : * * . * * * * * * * * : . . : * *

A1-MHA_0254 ACTGAGCGTGGCATTGTGTTATCCGCTCCACAAATTGATAAATTGCTACAGAAAACTAAA

B-COK_0274 ACTGAGCGTGGTATTGTTTTGTCAGCCCCTCAGCTTGATAAATTGCTTCAAAAAACCAAA

O-COI_0481 ACTGAGCGTGGCATTGTGTTATCCGCTCCACAAATTGATAAATTGCTACAGAAAACTAAA

DNA Homology *********** ***** ** ** ** ** ** ************* ** ***** ***

A1-MHA_0254 T E R G I V L S A P Q I D K L L Q K T K

B-COK_0274 T E R G I V L S A P Q L D K L L Q K T K

O-COI_0481 T E R G I V L S A P Q I D K L L Q K T K

Protein Homology * * * * * * * * * * * : * * * * * * * *

A1-MHA_0254 GCAGGCCAAGCATTAGGTTCTGCCGAAAGCATTGTACAAAATGCAAATAAAGCCAAAACT

B-COK_0274 GTAGGTCAAGCGATTGGCTCTACAGAAAATATTACAAAAGGTTTTAGTAATGCAAAAACA

O-COI_0481 GCAGGCCAAGCATTAGGTTCTGCCGAAAGCATTGTACAAAATGCAAATAAAGCCAAAACT

DNA Homology * *** ***** * ** *** * **** *** * ** * * *** ** *****

A1-MHA_0254 A G Q A L G S A E S I V Q N A N K A K T

B-COK_0274 V G Q A I G S T E N I T K G F S N A K T

O-COI_0481 A G Q A L G S A E S I V Q N A N K A K T

Protein Homology . * * * : * * : * . * . : . . : * * *

A1-MHA_0254 GTATTATCTGGCATTCAATCTATTTTAGGCTCAGTATTGGCTGGAATGGATTTAGATGAG

B-COK_0274 GTATTATCTGGAATCCAATCTATTCTAGGTTCTGTCTTAGCCGGTATGGACTTAGATGAA

O-COI_0481 GTATTATCTGGCATTCAATCTATTTTAGGCTCAGTATTGGCTGGAATGGATTTAGATGAG

DNA Homology *********** ** ********* **** ** ** ** ** ** ***** ********

A1-MHA_0254 V L S G I Q S I L G S V L A G M D L D E

B-COK_0274 V L S G I Q S I L G S V L A G M D L D E

O-COI_0481 V L S G I Q S I L G S V L A G M D L D E

Protein Homology * * * * * * * * * * * * * * * * * * * *

A1-MHA_0254 GCCTTACAGAATAACAGCAACCAACATGCTCTTGCTAAAGCTGGCTTGGAGCTAACAAAT

B-COK_0274 GCATTACAAAATAACAGCAATGAGCTAACACTTGCTAAAGCCGGTCTAGAGTTAACCAAC

O-COI_0481 GCCTTACAGAATAACAGCAACCAACATGCTCTTGCTAAAGCTGGCTTGGAGCTAACAAAT

DNA Homology ** ***** *********** * * * *********** ** * *** **** **

A1-MHA_0254 A L Q N N S N Q H A L A K A G L E L T N

B-COK_0274 A L Q N N S N E L T L A K A G L E L T N

O-COI_0481 A L Q N N S N Q H A L A K A G L E L T N

Protein Homology * * * * * * * : : * * * * * * * * * *

A1-MHA_0254 TCATTAATTGAAAATATTGCTAATTCAGTAAAAACACTTGACGAATTTGGTGAGCAAATT

B-COK_0274 TCGTTGATCGAAAATATTGCTAACTCAGTGAAAACATTAGACGCCTTTGGTGATCAAATT

O-COI_0481 TCATTAATTGAAAATATTGCTAATTCAGTAAAAACACTTGACGAATTTGGTGAGCAAATT

DNA Homology ** ** ** ************** ***** ****** * **** ******** ******

A1-MHA_0254 S L I E N I A N S V K T L D E F G E Q I

B-COK_0274 S L I E N I A N S V K T L D A F G D Q I

O-COI_0481 S L I E N I A N S V K T L D E F G E Q I

Protein Homology * * * * * * * * * * * * * * * * : * *

A1-MHA_0254 AGTCAATTTGGTTCAAAACTACAAAATATCAAAGGCTTAGGGACTTTAGGAGACAAACTC

B-COK_0274 AATCAACTTGGTTCAAAACTGCAAAATGTCAAAGGATTAAGCTCTCTTGGCGAAAAACTA

O-COI_0481 AGTCAATTTGGTTCAAAACTACAAAATATCAAAGGCTTAGGGACTTTAGGAGACAAACTC

DNA Homology * **** ************* ****** ******* *** * ** * ** ** *****

A1-MHA_0254 S Q F G S K L Q N I K G L G T L G D K L

B-COK_0274 N Q L G S K L Q N V K G L S S L G E K L

O-COI_0481 S Q F G S K L Q N I K G L G T L G D K L

Protein Homology . * : * * * * * * : * * * . : * * : * *

A1-MHA_0254 AAAAATATCGGTGGACTTGATAAAGCTGGCCTTGGTTTAGATGTTATCTCAGGGCTATTA

B-COK_0274 AAAGGTCTGAGTGGCTTTGATAAAACCAGCCTTGGTTTAGATATTGTATCCGGCTTGCTG

O-COI_0481 AAAAATATCGGTGGACTTGATAAAGCTGGCCTTGGTTTAGATGTTATCTCAGGGCTATTA

DNA Homology *** * * **** ******** * ************** ** * ** ** * *

A1-MHA_0254 K N I G G L D K A G L G L D V I S G L L

B-COK_0274 K G L S G F D K T S L G L D I V S G L L

O-COI_0481 K N I G G L D K A G L G L D V I S G L L

Protein Homology * . : . * : * * : . * * * * : : * * * *

A1-MHA_0254 TCGGGCGCAACAGCTGCACTTGTACTTGCAGATAAAAATGCTTCAACAGCTAAAAAAGTG

B-COK_0274 TCAGGAGCAACGGCTGCACTTGTACTTGCAGACAAAAACGCTTCAACATCAAGAAAAGTG

O-COI_0481 TCGGGCGCAACAGCTGCACTTGTACTTGCAGATAAAAATGCTTCAACAGCTAAAAAAGTG

DNA Homology ** ** ***** ******************** ***** ********* * * *******

A1-MHA_0254 S G A T A A L V L A D K N A S T A K K V

B-COK_0274 S G A T A A L V L A D K N A S T S R K V

O-COI_0481 S G A T A A L V L A D K N A S T A K K V

Protein Homology * * * * * * * * * * * * * * * * : : * *

A1-MHA_0254 GGTGCGGGTTTTGAATTGGCAAACCAAGTTGTTGGTAATATTACCAAAGCCGTTTCTTCT

B-COK_0274 GGTGCAGGTTTTGAATTAGCAAACCAAGTTGTTGGTAATATTACCAAAGCCGTTTCTTCT

O-COI_0481 GGTGCGGGTTTTGAATTGGCAAACCAAGTTGTTGGTAATATTACCAAAGCCGTTTCTTCT

DNA Homology ***** *********** ******************************************

A1-MHA_0254 G A G F E L A N Q V V G N I T K A V S S

B-COK_0274 G A G F E L A N Q V V G N I T K A V S S

O-COI_0481 G A G F E L A N Q V V G N I T K A V S S

Protein Homology * * * * * * * * * * * * * * * * * * * *

A1-MHA_0254 TACATTTTAGCCCAACGTGTTGCAGCAGGTTTATCTTCAACTGGGCCTGTGGCTGCTTTA

B-COK_0274 TACATTTTAGCCCAACGTGTTGCAGCCGGTTTATCTTCAACCGGACCTGTGGCAGCCTTA

O-COI_0481 TACATTTTAGCCCAACGTGTTGCAGCAGGTTTATCTTCAACTGGGCCTGTGGCTGCTTTA

DNA Homology ************************** ************** ** ******** ** ***

A1-MHA_0254 Y I L A Q R V A A G L S S T G P V A A L

B-COK_0274 Y I L A Q R V A A G L S S T G P V A A L

O-COI_0481 Y I L A Q R V A A G L S S T G P V A A L

Protein Homology * * * * * * * * * * * * * * * * * * * *

A1-MHA_0254 ATTGCTTCTACTGTTTCTCTTGCGATTAGCCCATTAGCATTTGCCGGTATTGCCGATAAA

B-COK_0274 ATTGCTTCTACCGTTTCTCTTGCGATTAGCCCATTATCATTTGCCGGTATTGCCGATAAA

O-COI_0481 ATTGCTTCTACTGTTTCTCTTGCGATTAGCCCATTAGCATTTGCCGGTATTGCCGATAAA

DNA Homology *********** ************************ ***********************

A1-MHA_0254 I A S T V S L A I S P L A F A G I A D K

B-COK_0274 I A S T V S L A I S P L S F A G I A D K

O-COI_0481 I A S T V S L A I S P L A F A G I A D K

Protein Homology * * * * * * * * * * * * : * * * * * * *

A1-MHA_0254 TTTAATCATGCAAAAAGTTTAGAGAGTTATGCCGAACGCTTTAAAAAATTAGGCTATGAC

B-COK_0274 TTTAATCACGCAAAAAGTTTAGAAAGCTATGCTGAACGTTTTAAAAAATTAGGCTATGAC

O-COI_0481 TTTAATCATGCAAAAAGTTTAGAGAGCTATGCCGAACGCTTTAAAAAATTAGGCTATGAC

DNA Homology ******** ************** ** ***** ***** *********************

A1-MHA_0254 F N H A K S L E S Y A E R F K K L G Y D

B-COK_0274 F N H A K S L E S Y A E R F K K L G Y D

O-COI_0481 F N H A K S L E S Y A E R F K K L G Y D

Protein Homology * * * * * * * * * * * * * * * * * * * *

A1-MHA_0254 GGAGATAATTTATTAGCAGAATATCAGCGGGGAACAGGGACTATTGATGCATCGGTTACT

B-COK_0274 GGTGATAATTTATTAGCAGAATATCAACGTGGAACAGGGACTATTGATGCTTCAGTAACA

O-COI_0481 GGAGATAATTTATTAGCAGAATATCAGCGGGGAACAGGGACTATTGATGCATCGGTTACT

DNA Homology ** *********************** ** ******************** ** ** **

A1-MHA_0254 G D N L L A E Y Q R G T G T I D A S V T

B-COK_0274 G D N L L A E Y Q R G T G T I D A S V T

O-COI_0481 G D N L L A E Y Q R G T G T I D A S V T

Protein Homology * * * * * * * * * * * * * * * * * * * *

A1-MHA_0254 GCAATTAATACCGCATTGGCCGCTATTGCTGGTGGTGTGTCTGCTGCTGCAGCCGGCTCG

B-COK_0274 GCAATCAATACAGCATTGGCTGCTATTGCTGGTGGTGTATCTGCCGCTGCTGCCGGCTCT

O-COI_0481 GCAATTAATACCGCATTGGCCGCTATTGCTGGTGGTGTGTCTGCTGCTGCAGCCGGCTCG

DNA Homology ***** ***** ******** ***************** ***** ***** ********

A1-MHA_0254 A I N T A L A A I A G G V S A A A A G S

B-COK_0274 A I N T A L A A I A G G V S A A A A G S

O-COI_0481 A I N T A L A A I A G G V S A A A A G S

Protein Homology * * * * * * * * * * * * * * * * * * * *

A1-MHA_0254 GTTATTGCTTCACCGATTGCCTTATTAGTATCTGGGATTACCGGTGTAATTTCTACGATT

B-COK_0274 GTGGTTGCTTCACCAATTGCCCTACTTGTATCAGGTATTACCGGTGTGATTTCCACTATT

O-COI_0481 GTTATTGCTTCACCGATTGCCTTATTAGTATCTGGGATTACCGGTGTAATTTCTACGATT

DNA Homology ** ********** ****** ** * ***** ** *********** ***** ** ***

A1-MHA_0254 V I A S P I A L L V S G I T G V I S T I

B-COK_0274 V V A S P I A L L V S G I T G V I S T I

O-COI_0481 V I A S P I A L L V S G I T G V I S T I

Protein Homology * : * * * * * * * * * * * * * * * * * *

A1-MHA_0254 CTGCAATATTCTAAACAAGCAATGTTTGAGCACGTTGCAAATAAAATTCATAACAAAATT

B-COK_0274 CTACAGTACTCTAAACAAGCAATGTTTGAACACGTTGCAAATAAAATTCACAATAAAATT

O-COI_0481 CTGCAATATTCTAAACAAGCAATGTTTGAGCACGTTGCAAATAAAATTCATAACAAAATT

DNA Homology ** ** ** ******************** ******************** ** ******

A1-MHA_0254 L Q Y S K Q A M F E H V A N K I H N K I

B-COK_0274 L Q Y S K Q A M F E H V A N K I H N K I

O-COI_0481 L Q Y S K Q A M F E H V A N K I H N K I

Protein Homology * * * * * * * * * * * * * * * * * * * *

A1-MHA_0254 GTAGAATGGGAAAAAAATAATCACGGTAAGAACTACTTTGAAAATGGTTACGATGCCCGT

B-COK_0274 GTAGAATGGGAAAAAAATAATCCAGGTAAGAACTACTTTGAAAATGGTTACGATGCCCGT

O-COI_0481 GTAGAATGGGAAAAAAATAATCACGGTAAGAACTACTTTGAAAATGGTTACGATGCCCGT

DNA Homology ********************** ************************************

A1-MHA_0254 V E W E K N N H G K N Y F E N G Y D A R

B-COK_0274 V E W E K N N P G K N Y F E N G Y D A R

O-COI_0481 V E W E K N N H G K N Y F E N G Y D A R

Protein Homology * * * * * * * * * * * * * * * * * * *

A1-MHA_0254 TATCTTGCGAATTTACAAGATAATATGAAATTCTTACTGAACTTAAACAAAGAGTTACAG

B-COK_0274 TATCTTGCGAATTTACAAGATAATATGAAATTTTTACTGAACTTAAACAAAGAGTTACAG

O-COI_0481 TATCTTGCGAATTTACAAGATAATATGAAATTCTTACTGAACTTAAACAAAGAGTTACAG

DNA Homology ******************************** ***************************

A1-MHA_0254 Y L A N L Q D N M K F L L N L N K E L Q

B-COK_0274 Y L A N L Q D N M K F L L N L N K E L Q

O-COI_0481 Y L A N L Q D N M K F L L N L N K E L Q

Protein Homology * * * * * * * * * * * * * * * * * * * *

A1-MHA_0254 GCAGAACGTGTCATCGCTATTACTCAGCAGCAATGGGATAACAACATTGGTGATTTAGCT

B-COK_0274 GCAGAACGTGTTATTGCAATTACTCAGCAGCAATGGGATAACAACATTGGTGATTTAGCC

O-COI_0481 GCAGAACGTGTCATCGCTATTACTCAGCAGCAATGGGATAACAACATTGGTGATTTAGCT

DNA Homology *********** ** ** *****************************************

A1-MHA_0254 A E R V I A I T Q Q Q W D N N I G D L A

B-COK_0274 A E R V I A I T Q Q Q W D N N I G D L A

O-COI_0481 A E R V I A I T Q Q Q W D N N I G D L A

Protein Homology * * * * * * * * * * * * * * * * * * * *

A1-MHA_0254 GGTATTAGCCGTTTAGGTGAAAAAGTCCTTAGTGGTAAAGCCTATGTGGATGCGTTTGAA

B-COK_0274 GGTATCAGCCGTTTAGGTGAAAAAGTCCTTAGTGGTAAAGCCTATGTGGATGCGTTTGAA

O-COI_0481 GGTATTAGCCGTTTAGGTGAAAAAGTCCTTAGTGGTAAAGCCTATGTGGATGCGTTTGAA

DNA Homology ***** ******************************************************

A1-MHA_0254 G I S R L G E K V L S G K A Y V D A F E

B-COK_0274 G I S R L G E K V L S G K A Y V D A F E

O-COI_0481 G I S R L G E K V L S G K A Y V D A F E

Protein Homology * * * * * * * * * * * * * * * * * * * *

A1-MHA_0254 GAAGGCAAACACATTAAAGCCGATAAATTAGTACAGTTGGATTCGGCAAACGGTATTATT

B-COK_0274 GAAGGTAAACACCTTAAAGCTGATAAATTAGTACAGTTAGATTCGGCAAACGGTATTATT

O-COI_0481 GAAGGCAAACACATTAAAGCCGATAAATTAGTACAGTTGGATTCGGCAAACGGTATTATT

DNA Homology ***** ****** ******* ***************** *********************

A1-MHA_0254 E G K H I K A D K L V Q L D S A N G I I

B-COK_0274 E G K H L K A D K L V Q L D S A N G I I

O-COI_0481 E G K H I K A D K L V Q L D S A N G I I

Protein Homology * * * * : * * * * * * * * * * * * * * *

A1-MHA_0254 GATGTGAGTAATTCGGGTAAAGCGAAAACTCAGCATATCTTATTCAGAACGCCATTATTG

B-COK_0274 GATGTGAGTAATTCAGGTAAAGCGAAAACTCAACATATTTTATTCCGAACGCCACTACTA

O-COI_0481 GATGTGAGTAATTCGGGTAAAGCGAAAACTCAGCATATCTTATTCAGAACGCCATTATTG

DNA Homology ************** ***************** ***** ****** ******** ** *

A1-MHA_0254 D V S N S G K A K T Q H I L F R T P L L

B-COK_0274 D V S N S G K A K T Q H I L F R T P L L

O-COI_0481 D V S N S G K A K T Q H I L F R T P L L

Protein Homology * * * * * * * * * * * * * * * * * * * *

A1-MHA_0254 ACGCCGGGAACAGAGCATCGTGAACGCGTACAAACAGGTAAATATGAATATATTACCAAG

B-COK_0274 ACACCGGGTACAGAAAAACGTGAACGTGTACAAACCGGTAAGTATGAATATATTACTAAA

O-COI_0481 ACGCCGGGAACAGAGCATCGTGAACGCGTACAAACAGGTAAATATGAATATATTACCAAG

DNA Homology ** ***** ***** * ******** ******** ***** ************** **

A1-MHA_0254 T P G T E H R E R V Q T G K Y E Y I T K

B-COK_0274 T P G T E K R E R V Q T G K Y E Y I T K

O-COI_0481 T P G T E H R E R V Q T G K Y E Y I T K

Protein Homology * * * * * : * * * * * * * * * * * * * *

A1-MHA_0254 CTCAATATTAACCGTGTAGATAGCTGGAAAATTACAGATGGTGCAGCAAGTTCTACCTTT

B-COK_0274 CTCAATATTAACCGTGTAGATAGCTGGAAGATCACAGATGGTGAAGCAAGCTCTACATTT

O-COI_0481 CTCAATATTAACCGTGTAGATAGCTGGAAAATTACAGATGGTGCAGCAAGTTCTACCTTT

DNA Homology ***************************** ** ********** ****** ***** ***

A1-MHA_0254 L N I N R V D S W K I T D G A A S S T F

B-COK_0274 L N I N R V D S W K I T D G E A S S T F

O-COI_0481 L N I N R V D S W K I T D G A A S S T F

Protein Homology * * * * * * * * * * * * * * * * * * *

A1-MHA_0254 GATTTAACTAACGTTGTTCAGCGTATTGGTATTGAATTAGACAATGCTGGAAATGTAACT

B-COK_0274 GATTTAACTAATGTTGTTCAACGTATCGGTATTGAATTAGACAATGCTGGAAATGTAACT

O-COI_0481 GATTTAACTAACGTTGTTCAGCGTATTGGTATTGAATTAGACAATGCTGGAAATGTAACT

DNA Homology *********** ******** ***** *********************************

A1-MHA_0254 D L T N V V Q R I G I E L D N A G N V T

B-COK_0274 D L T N V V Q R I G I E L D N A G N V T

O-COI_0481 D L T N V V Q R I G I E L D N A G N V T

Protein Homology * * * * * * * * * * * * * * * * * * * *

A1-MHA_0254 AAAACCAAAGAAACAAAAATTATTGCCAAACTTGGTGAAGGTGATGACAACGTATTTGTT

B-COK_0274 AAAACCAAAGAAACAAAAATTATTGCCAAACTTGGTGAAGGTGATGACAACGTATTTGTT

O-COI_0481 AAAACCAAAGAAACAAAAATTATTGCCAAACTTGGTGAAGGTGATGACAACGTATTTGTT

DNA Homology ************************************************************

A1-MHA_0254 K T K E T K I I A K L G E G D D N V F V

B-COK_0274 K T K E T K I I A K L G E G D D N V F V

O-COI_0481 K T K E T K I I A K L G E G D D N V F V

Protein Homology * * * * * * * * * * * * * * * * * * * *

A1-MHA_0254 GGTTCTGGTACGACGGAAATTGATGGCGGTGAAGGTTACGACCGAGTTCACTATAGCCGT

B-COK_0274 GGTTCTGGTACGACGGAAATTGATGGCGGTGAAGGTTACGACCGAGTTCACTATAGCCGT

O-COI_0481 GGTTCTGGTACGACGGAAATTGATGGCGGTGAAGGTTACGACCGAGTTCACTATAGCCGT

DNA Homology ************************************************************

A1-MHA_0254 G S G T T E I D G G E G Y D R V H Y S R

B-COK_0274 G S G T T E I D G G E G Y D R V H Y S R

O-COI_0481 G S G T T E I D G G E G Y D R V H Y S R

Protein Homology * * * * * * * * * * * * * * * * * * * *

A1-MHA_0254 GGAAACTATGGTGCTTTAACTATTGATGCAACCAAAGAGACCGAGCAAGGTAGTTATACC

B-COK_0274 GGAAACTATGGTGCTTTAACTATTGATGCAACCAAAGAGACCGAGCAAGGTAGTTATACC

O-COI_0481 GGAAACTATGGTGCTTTAACTATTGATGCAACCAAAGAGACCGAGCAAGGTAGTTATACC

DNA Homology ************************************************************

A1-MHA_0254 G N Y G A L T I D A T K E T E Q G S Y T

B-COK_0274 G N Y G A L T I D A T K E T E Q G S Y T

O-COI_0481 G N Y G A L T I D A T K E T E Q G S Y T

Protein Homology * * * * * * * * * * * * * * * * * * * *

A1-MHA_0254 GTAAATCGTTTCGTAGAAACCGGTAAAGCACTACACGAAGTGACTTCAACCCATACCGCA

B-COK_0274 GTAAATCGTTTCGTAGAAACCGGTAAAGCACTACACGAAGTGACTTCAACCCATACTGCA

O-COI_0481 GTAAATCGTTTCGTAGAAACCGGTAAAGCACTACACGAAGTGACTTCAACCCATACCGCA

DNA Homology ******************************************************** ***

A1-MHA_0254 V N R F V E T G K A L H E V T S T H T A

B-COK_0274 V N R F V E T G K A L H E V T S T H T A

O-COI_0481 V N R F V E T G K A L H E V T S T H T A

Protein Homology * * * * * * * * * * * * * * * * * * * *

A1-MHA_0254 TTAGTGGGCAACCGTGAAGAAAAAATAGAATATCGTCATAGCAATAACCAGCACCATGCC

B-COK_0274 TTAGTTGGCAGCAGAGAAGAAAAAATTGAGTATCGCCATAGTAATAATAGACAGCATGCC

O-COI_0481 TTAGTGGGCAACCGTGAAGAAAAAATAGAATATCGTCATAGCAATAACCAGCACCATGCC

DNA Homology ***** **** * * *********** ** ***** ***** ***** ** ******

A1-MHA_0254 L V G N R E E K I E Y R H S N N Q H H A

B-COK_0274 L V G S R E E K I E Y R H S N N R Q H A

O-COI_0481 L V G N R E E K I E Y R H S N N Q H H A

Protein Homology * * * . * * * * * * * * * * * * : : * *

A1-MHA_0254 GGTTATTACACCAAAGATACCTTGAAAGCTGTTGAAGAAATTATCGGTACATCACATAAC

B-COK_0274 GGTTATTATACAAAAGATACACTAACATCTATTGAAGAAATTATTGGTACATCACACAAT

O-COI_0481 GGTTATTACACCAAAGATACCTTGAAAGCTGTTGAAGAAATTATCGGTACATCACATAAC

DNA Homology ******** ** ******** * * * ** ************* *********** **

A1-MHA_0254 G Y Y T K D T L K A V E E I I G T S H N

B-COK_0274 G Y Y T K D T L T S I E E I I G T S H N

O-COI_0481 G Y Y T K D T L K A V E E I I G T S H N

Protein Homology * * * * * * * * . : : * * * * * * * * *

A1-MHA_0254 GATATCTTTAAAGGTAGTAAGTTCAATGATGCCTTTAACGGTGGTGATGGTGTCGATACT

B-COK_0274 GATATCTTCAAAGGTAGTCAGTTCAATGATGCCTTTAACGGTGGGGATGGTGTTGATACT

O-COI_0481 GATATCTTTAAAGGTAGTAAGTTCAATGATGCCTTTAACGGTGGTGATGGTGTCGATACT

DNA Homology ******** ********* ************************* ******** ******

A1-MHA_0254 D I F K G S K F N D A F N G G D G V D T

B-COK_0274 D I F K G S Q F N D A F N G G D G V D T

O-COI_0481 D I F K G S K F N D A F N G G D G V D T

Protein Homology * * * * * * : * * * * * * * * * * * * *

A1-MHA_0254 ATTGACGGTAACGACGGCAATGACCGCTTATTTGGTGGTAAAGGCGATGATATTCTCGAT

B-COK_0274 ATTGACGGCAATGGAGGTAATGACCGCTTATTTGGTGGTAAAGGTGATGATATTATTGAT

O-COI_0481 ATTGACGGTAACGACGGCAATGACCGCTTATTTGGTGGTAAAGGCGATGATATTCTCGAT

DNA Homology ******** ** * ** ************************** ********* * ***

A1-MHA_0254 I D G N D G N D R L F G G K G D D I L D

B-COK_0274 I D G N G G N D R L F G G K G D D I I D

O-COI_0481 I D G N D G N D R L F G G K G D D I L D

Protein Homology * * * * . * * * * * * * * * * * * * : *

A1-MHA_0254 GGTGGAAATGGTGATGATTTTATCGATGGCGGTAAAGGCAACGACCTATTACACGGTGGC

B-COK_0274 GGTGGAGACGGTGATGACTTTATCGATGGCGGTAAAGGCAATGACCTCTTACACGGCGGC

O-COI_0481 GGTGGAAATGGTGATGATTTTATCGATGGCGGTAAAGGCAACGACCTATTACACGGTGGC

DNA Homology ****** * ******** *********************** ***** ******** ***

A1-MHA_0254 G G N G D D F I D G G K G N D L L H G G

B-COK_0274 G G D G D D F I D G G K G N D L L H G G

O-COI_0481 G G N G D D F I D G G K G N D L L H G G

Protein Homology * * : * * * * * * * * * * * * * * * * *

A1-MHA_0254 AAGGGCGATGATATTTTCGTTCACCGTAAAGGCGATGGTAATGATATTATTACCGATTCT

B-COK_0274 AGAGGCGATGATATTTTCGTTCACCGCCAAGGCGATGGTAATGATTCGATTACTGAAGCT

O-COI_0481 AAGGGCGATGATATTTTCGTTCACCGTAAAGGCGATGGTAATGATATTATTACCGATTCT

DNA Homology * *********************** ***************** ***** ** **

A1-MHA_0254 K G D D I F V H R K G D G N D I I T D S

B-COK_0274 R G D D I F V H R Q G D G N D S I T E A

O-COI_0481 K G D D I F V H R K G D G N D I I T D S

Protein Homology : * * * * * * * * : * * * * * * * : :

A1-MHA_0254 GACGGCAATGATAAATTATCATTCTCTGATTCGAACTTAAAAGATTTAACATTTGAAAAA

B-COK_0274 GGGGGACACGACAGACTCTCTTTCTCAGATTCAAATCTAAAAGATTTAACCTTCGAGAAA

O-COI_0481 GACGGCAATGATAAATTATCATTCTCTGATTCGAACTTAAAAGATTTAACATTTGAAAAA

DNA Homology * ** * ** * * * ** ***** ***** ** ************* ** ** ***

A1-MHA_0254 D G N D K L S F S D S N L K D L T F E K

B-COK_0274 G G H D R L S F S D S N L K D L T F E K

O-COI_0481 D G N D K L S F S D S N L K D L T F E K

Protein Homology . * : * : * * * * * * * * * * * * * * *

A1-MHA_0254 GTTAAACATAATCTTGTCATCACGAATAGCAAAAAAGAGAAAGTGACCATTCAAAACTGG

B-COK_0274 GTAAATCATCATCTTGTGATTACCAACACTAAGCAGGAAAAAGTCACTATTCAGAACTGG

O-COI_0481 GTTAAACATAATCTTGTCATCACGAATAGCAAAAAAGAGAAAGTGACCATTCAAGACTGG

DNA Homology ** ** *** ******* ** ** ** * ** * ** ***** ** ***** *****

A1-MHA_0254 V K H N L V I T N S K K E K V T I Q N W

B-COK_0274 V N H H L V I T N T K Q E K V T I Q N W

O-COI_0481 V K H N L V I T N S K K E K V T I Q D W

Protein Homology * : * : * * * * * : * : * * * * * * : *

A1-MHA_0254 TTCCGAGAGGCTGATTTTGCTAAAGAAGTGCCTAATTATAAAGCAACTAAAGATGAGAAA

B-COK_0274 TTCCGTGAAGAAGAATTTGCGAAAACAGTTAAAAATTATGTTGCCACTAGAGATGAAAAA

O-COI_0481 TTCCGAGAGGCTGATTTTGCTAAAGAAGTGCCTAATTATAAAGCAACTAAAGATGAGAAA

DNA Homology ***** ** * ** ***** *** *** ****** ** **** ****** ***

A1-MHA_0254 F R E A D F A K E V P N Y K A T K D E K

B-COK_0274 F R E E E F A K T V K N Y V A T R D E K

O-COI_0481 F R E A D F A K E V P N Y K A T K D E K

Protein Homology * * * : * * * * * * * * : * * *

A1-MHA_0254 ATCGAAGAAATCATCGGTCAAAATGGCGAGCGGATCACCTCAAAGCAAGTTGATGATCTT

B-COK_0274 ATCGAAGAAATCATCGGGCAAAATGGTGAGCGGATTACCTCGAAGCAAGTTGATGAGCTT

O-COI_0481 ATCGAAGAAATCATCGGTCAAAATGGCGAGCGGATCACCTCAAAGCAAGTTGATGATCTT

DNA Homology ***************** ******** ******** ***** ************** ***

A1-MHA_0254 I E E I I G Q N G E R I T S K Q V D D L

B-COK_0274 I E E I I G Q N G E R I T S K Q V D E L

O-COI_0481 I E E I I G Q N G E R I T S K Q V D D L

Protein Homology * * * * * * * * * * * * * * * * * * : *

A1-MHA_0254 ATCGCAAAAGGTAACGGCAA---AATTACCCAAGATGAGCTATCAAAAGTTGTTGATAAC

B-COK_0274 ATTGCAAAAGGTAAAGATAATAAAATTGATAAAAATGATTTGGCAAATGTGGTTAACTCC

O-COI_0481 ATCGCAAAAGGTAACGGCAA---AATTACCCAAGATGAGCTATCAAAAGTTGTTGATAAC

DNA Homology ** *********** * ** **** ** **** * **** ** *** * *

A1-MHA_0254 I A K G N G - K I T Q D E L S K V V D N

B-COK_0274 I A K G K D N K I D K N D L A N V V N S

O-COI_0481 I A K G N G - K I T Q D E L S K V V D N

Protein Homology * * * * : . * * : : : * : : * * : .

A1-MHA_0254 TATGAATTGCTCAAACATAGCAAAAATGTGACAAACAGCTTAGATAAGTTAATCTCATCT

B-COK_0274 TATGAATTACTCAAGAATAGCCGAAATGTAACAAACAGCTTAGATAAATTAATTTCATCT

O-COI_0481 TATGAATTGCTCAAACATAGCAAAAATGTGACAAACAGCTTAGATAAGTTAATCTCATCT

DNA Homology ******** ***** ***** ****** ***************** ***** ******

A1-MHA_0254 Y E L L K H S K N V T N S L D K L I S S

B-COK_0274 Y E L L K N S R N V T N S L D K L I S S

O-COI_0481 Y E L L K H S K N V T N S L D K L I S S

Protein Homology * * * * * : * : * * * * * * * * * * * *

A1-MHA_0254 GTAAGTGCATTTACCTCGTCTAATGATTCGAGAAATGTATTAGTGGCTCCAACTTCAATG

B-COK_0274 GTGAGCTCGTTTACTTCGTCTAATGATTCGAGAAATGTATTAGCGACTCCAACTTCAATG

O-COI_0481 GTAAGTGCATTTACCTCGTCTAATGATTCGAGAAATGTATTAGTGGCTCCAACTTCAATG

DNA Homology ** ** * ***** **************************** * **************

A1-MHA_0254 V S A F T S S N D S R N V L V A P T S M

B-COK_0274 V S S F T S S N D S R N V L A T P T S M

O-COI_0481 V S A F T S S N D S R N V L V A P T S M

Protein Homology * * : * * * * * * * * * * * . : * * * *

A1-MHA_0254 TTGGATCAAAGTTTATCTTCTCTTCAATTTGCTAGAGCAGCT

B-COK_0274 CTGGATACAAGTTTATCTTCTCTACAATTTGCTAGAGCAGCT

O-COI_0481 TTGGATCAAAGTTTATCTTCTCTTCAATTTGCTAGAGCAGCT

DNA Homology ***** *************** ******************

A1-MHA_0254 L D Q S L S S L Q F A R A A

B-COK_0274 L D T S L S S L Q F A R A A

O-COI_0481 L D Q S L S S L Q F A R A A

Protein Homology * * * * * * * * * * * * *

Multiple sequence alignment of LktA DNA and protein sequences.

A1-MHA_0255 ------------------------------------ATGGAAGCTAACCATCAAAGGAAT

B-COK_0273 ATGGAGTTGCCGTTTTATTTTATAAAAGGAGACAATATGGAAGCTAATCATCAAAGGAAT

O-COI_0482 ------------------------------------ATGGAAGCTAACCATCAAAGGAAT

DNA Homology *********** ************

A1-MHA_0255 - - - - - - - - - - - - M E A N H Q R N

B-COK_0273 M E L P F Y F I K G D N M E A N H Q R N

O-COI_0482 - - - - - - - - - - - - M E A N H Q R N

Protein Homology * * * * * * * *

A1-MHA_0255 GATCTTGGTTTAGTTGCCCTCACTATGTTGGCACAATACCATAATATTTCGCTTAATCCG

B-COK_0273 GATCTTGGTTTAGTAGCCCTCACCATGTTGGCACAATACCATAATATTTCGCTTAATCCG

O-COI_0482 GATCTTGGTTTAGTTGCCCTCACTATGTTGGCACAATACCATAATATTTCGCTTAATCCG

DNA Homology ************** ******** ************************************

A1-MHA_0255 D L G L V A L T M L A Q Y H N I S L N P

B-COK_0273 D L G L V A L T M L A Q Y H N I S L N P

O-COI_0482 D L G L V A L T M L A Q Y H N I S L N P

Protein Homology * * * * * * * * * * * * * * * * * * * *

A1-MHA_0255 GAAGAAATAAAACATAAATTTGATCTTGACGGAAAAGGGCTTTCTTTAACTGCTTGGCTT

B-COK_0273 GAAGAAATAAAACATAAGTTTGATCTTGACGGGAAAGGGCTTTCTTTAACTGCTTGGCTT

O-COI_0482 GAAGAAATAAAACATAAATTTGATCTTGACGGAAAAGGGCTTTCTTTAACTGCTTGGCTT

DNA Homology ***************** ************** ***************************

A1-MHA_0255 E E I K H K F D L D G K G L S L T A W L

B-COK_0273 E E I K H K F D L D G K G L S L T A W L

O-COI_0482 E E I K H K F D L D G K G L S L T A W L

Protein Homology * * * * * * * * * * * * * * * * * * * *

A1-MHA_0255 TTAGCTGCAAAATCGTTAGCGTTGAAAGCGAAACACATTAAAAAAGAGATTTCCCGCTTA

B-COK_0273 TTAGCTGCAAAATCATTGGCGTTGAAAGCAAAACATATTAAAAAAGAGGTTTCCCGCTTA

O-COI_0482 TTAGCTGCAAAATCGTTAGCGTTGAAAGCGAAACACATTAAAAAAGAGATTTCCCGCTTA

DNA Homology ************** ** *********** ***** ************ ***********

A1-MHA_0255 L A A K S L A L K A K H I K K E I S R L

B-COK_0273 L A A K S L A L K A K H I K K E V S R L

O-COI_0482 L A A K S L A L K A K H I K K E I S R L

Protein Homology * * * * * * * * * * * * * * * * : * * *

A1-MHA_0255 CACTTGGTGAATTTACCGGCATTAGTTTGGCAAGATAACGGTAAACATTTTTTATTGGTA

B-COK_0273 CATTTGGTTAATTTACCGGCATTAGTTTGGCAAGATAACGGCAAACATTTTTTGCTGGTA

O-COI_0482 CACTTGGTGAATTTACCGGCATTAGTTTGGCAAGATAACGGTAAACATTTTTTATTGGTA

DNA Homology ** ***** ******************************** *********** *****

A1-MHA_0255 H L V N L P A L V W Q D N G K H F L L V

B-COK_0273 H L V N L P A L V W Q D N G K H F L L V

O-COI_0482 H L V N L P A L V W Q D N G K H F L L V

Protein Homology * * * * * * * * * * * * * * * * * * * *

A1-MHA_0255 AAAGTGGATACCGATAATAACCGCTATTTAACTTACAATTTGGAACAAGATGCTCCACAA

B-COK_0273 AAAGTAGATACCGATAATAACCGCTATTTAACCTACGATTTAGAAAAAGATGCTCCACAA

O-COI_0482 AAAGTGGATACCGATAATAACCGCTATTTAACTTACAATTTGGAACAAGATGCTCCACAA

DNA Homology ***** ************************** *** **** *** **************

A1-MHA_0255 K V D T D N N R Y L T Y N L E Q D A P Q

B-COK_0273 K V D T D N N R Y L T Y D L E K D A P Q

O-COI_0482 K V D T D N N R Y L T Y N L E Q D A P Q

Protein Homology * * * * * * * * * * * * : * * : * * * *

A1-MHA_0255 ATTCTGTCACAAGACGAATTTGAAGCCTGCTATCAAGGGCAGTTAATTTTGGTCACGTCC

B-COK_0273 ATTCTGTCACAAGATGAGTTTGAAGCCTGCTATCAAGGGCAATTAATTCTGGTTACCTCC

O-COI_0482 ATTCTGTCACAAGACGAATTTGAAGCCTGCTATCAAGGGCAGTTAATTTTGGTCACGTCC

DNA Homology ************** ** *********************** ****** **** ** ***

A1-MHA_0255 I L S Q D E F E A C Y Q G Q L I L V T S

B-COK_0273 I L S Q D E F E A C Y Q G Q L I L V T S

O-COI_0482 I L S Q D E F E A C Y Q G Q L I L V T S

Protein Homology * * * * * * * * * * * * * * * * * * * *

A1-MHA_0255 AGAGCTTCCGTAGTAGGTCAATTAGCAAAGTTCGATTTCACCTGGTTTATTCCGGCGGTG

B-COK_0273 AGAGCTTCCGTAGTAGGTCAATTAGCAAAGTTCGATTTCACTTGGTTTATCCCGGCTGTG

O-COI_0482 AGAGCTTCCGTAGTAGGTCAATTAGCAAAGTTCGATTTCACCTGGTTTATTCCGGCGGTG

DNA Homology ***************************************** ******** ***** ***

A1-MHA_0255 R A S V V G Q L A K F D F T W F I P A V

B-COK_0273 R A S V V G Q L A K F D F T W F I P A V

O-COI_0482 R A S V V G Q L A K F D F T W F I P A V

Protein Homology * * * * * * * * * * * * * * * * * * * *

A1-MHA_0255 ATCAAATACCGAAAAATCTTTCTAGAAACCTTGATTGTTTCGATCTTTTTGCAAATTTTT

B-COK_0273 ATCAAATATCGAAAAATCTTTCTAGAAACCTTGATTGTTTCGATCTTTTTGCAAATTTTT

O-COI_0482 ATCAAATACCGAAAAATCTTTCTAGAAACCTTGATTGTTTCGATCTTTTTGCAAATTTTT

DNA Homology ******** ***************************************************

A1-MHA_0255 I K Y R K I F L E T L I V S I F L Q I F

B-COK_0273 I K Y R K I F L E T L I V S I F L Q I F

O-COI_0482 I K Y R K I F L E T L I V S I F L Q I F

Protein Homology * * * * * * * * * * * * * * * * * * * *

A1-MHA_0255 GCCCTAATTACACCGCTATTCTTCCAAGTTGTTATGGATAAAGTACTGGTGCATCGAGGT

B-COK_0273 GCCCTAATTACACCGCTATTCTTCCAAGTTGTTATGGATAAAGTACTGGTGCATCGAGGT

O-COI_0482 GCCCTAATTACACCGCTATTCTTCCAAGTTGTTATGGATAAAGTACTGGTGCATCGAGGT

DNA Homology ************************************************************

A1-MHA_0255 A L I T P L F F Q V V M D K V L V H R G

B-COK_0273 A L I T P L F F Q V V M D K V L V H R G

O-COI_0482 A L I T P L F F Q V V M D K V L V H R G

Protein Homology * * * * * * * * * * * * * * * * * * * *

A1-MHA_0255 TTTTCAACCTTGAATATCATTACGGTTGCCTTAGCTATTGTGATCATCTTTGAAATTGTA

B-COK_0273 TTTTCAACCTTGAATATCATTACGGTTGCCTTAGCTATTGTGATCATCTTTGAAATTGTC

O-COI_0482 TTTTCAACCTTGAATATCATTACGGTTGCCTTAGCTATTGTGATCATCTTTGAAATTGTA

DNA Homology ***********************************************************

A1-MHA_0255 F S T L N I I T V A L A I V I I F E I V

B-COK_0273 F S T L N I I T V A L A I V I I F E I V

O-COI_0482 F S T L N I I T V A L A I V I I F E I V

Protein Homology * * * * * * * * * * * * * * * * * * * *

A1-MHA_0255 CTAAGTGGTTTGAGAACCTATGTTTTTTCTCATAGCACTAGCCGTATTGATGTTGAATTA

B-COK_0273 TTAAGCGGTTTACGCACCTACGTGTTTTCCCATAGCACTAGTCGGATTGACGTGGAATTA

O-COI_0482 CTAAGTGGTTTGAGAACCTATGTTTTTTCTCATAGCACTAGCCGTATTGATGTTGAATTA

DNA Homology **** ***** * ***** ** ***** *********** ** ***** ** ******

A1-MHA_0255 L S G L R T Y V F S H S T S R I D V E L

B-COK_0273 L S G L R T Y V F S H S T S R I D V E L

O-COI_0482 L S G L R T Y V F S H S T S R I D V E L

Protein Homology * * * * * * * * * * * * * * * * * * * *

A1-MHA_0255 GGCGCTAAATTATTTCGACATTTATTATCACTACCCATTTCTTATTTTGAAAACAGACGA

B-COK_0273 GGAGCTAAATTATTTAGGCATTTATTATCGTTACCTATTTCTTATTTTGAGAACAGACGT

O-COI_0482 GGCGCTAAATTATTTCGACATTTATTATCACTACCCATTTCTTATTTTGAAAACAGACGA

DNA Homology ** ************ * *********** **** ************** ********

A1-MHA_0255 G A K L F R H L L S L P I S Y F E N R R

B-COK_0273 G A K L F R H L L S L P I S Y F E N R R

O-COI_0482 G A K L F R H L L S L P I S Y F E N R R

Protein Homology * * * * * * * * * * * * * * * * * * * *

A1-MHA_0255 GTTGGAGATACAGTCGCTAGGGTTAGAGAATTAGATCAAATTCGTAATTTCCTTACCGGA

B-COK_0273 GTTGGCGATACAGTTGCACGGGTCAGAGAATTGGATCAAATACGAAATTTCCTTACCGGA

O-COI_0482 GTTGGAGATACAGTCGCTAGGGTTAGAGAATTAGATCAAATTCGTAATTTCCTTACCGGA

DNA Homology ***** ******** ** **** ******** ******** ** ***************

A1-MHA_0255 V G D T V A R V R E L D Q I R N F L T G

B-COK_0273 V G D T V A R V R E L D Q I R N F L T G

O-COI_0482 V G D T V A R V R E L D Q I R N F L T G

Protein Homology * * * * * * * * * * * * * * * * * * * *

A1-MHA_0255 CAAGCATTAACCTCGGTGTTAGATCTCTTATTCTCTTTTATCTTTTTTGCCGTAATGTGG

B-COK_0273 CAAGCATTAACCTCGGTATTAGATTTATTATTCTCTTTTATCTTTTTTGCCGTAATGTGG

O-COI_0482 CAAGCATTAACCTCGGTGTTAGATCTCTTATTCTCTTTTATCTTTTTTGCCGTAATGTGG

DNA Homology ***************** ****** * *********************************

A1-MHA_0255 Q A L T S V L D L L F S F I F F A V M W

B-COK_0273 Q A L T S V L D L L F S F I F F A V M W

O-COI_0482 Q A L T S V L D L L F S F I F F A V M W

Protein Homology * * * * * * * * * * * * * * * * * * * *

A1-MHA_0255 TATTACAGCCCAAAATTAACCTTGGTAATTCTTGGTTCATTGCCCTGCTATATTTTATGG

B-COK_0273 TATTACAGCCCGAAATTGACCTTAGTCATTCTCGGCTCACTACCCTGCTATATTTTATGG

O-COI_0482 TATTACAGCCCAAAATTAACCTTGGTAATTCTTGGTTCATTGCCCTGCTATATTTTATGG

DNA Homology *********** ***** ***** ** ***** ** *** * ******************

A1-MHA_0255 Y Y S P K L T L V I L G S L P C Y I L W

B-COK_0273 Y Y S P K L T L V I L G S L P C Y I L W

O-COI_0482 Y Y S P K L T L V I L G S L P C Y I L W

Protein Homology * * * * * * * * * * * * * * * * * * * *

A1-MHA_0255 TCAATTTTTATTAGTCCGATTTTAAGACGGCGTTTAGATGAGAAATTTGCCCGAAGTGCT

B-COK_0273 TCAATTTTTATTAGTCCGATTTTAAGACGGCGTTTAGACGATAAATTTGCCCGAAGTGCT

O-COI_0482 TCAATTTTTATTAGTCCGATTTTAAGACGGCGTTTAGATGAGAAATTTGCCCGAAGTGCT

DNA Homology ************************************** ** ******************

A1-MHA_0255 S I F I S P I L R R R L D E K F A R S A

B-COK_0273 S I F I S P I L R R R L D D K F A R S A

O-COI_0482 S I F I S P I L R R R L D E K F A R S A

Protein Homology * * * * * * * * * * * * * : * * * * * *

A1-MHA_0255 GATAACCAAGCATTCTTAGTTGAGTCGGTAACAGCCATCAATATGATTAAAGCGATGGCG

B-COK_0273 GACAATCAAGCATTCTTAGTTGAATCGGTAACAGCCATCAATATGATTAAAGCGATGGCG

O-COI_0482 GATAACCAAGCATTCTTAGTTGAGTCGGTAACAGCCATCAATATGATTAAAGCGATGGCG

DNA Homology ** ** ***************** ************************************

A1-MHA_0255 D N Q A F L V E S V T A I N M I K A M A

B-COK_0273 D N Q A F L V E S V T A I N M I K A M A

O-COI_0482 D N Q A F L V E S V T A I N M I K A M A

Protein Homology * * * * * * * * * * * * * * * * * * * *

A1-MHA_0255 GTTGCTCCACAAATGACGGATACATGGGATAAACAGCTGGCAAGCTATGTTTCATCAAGT

B-COK_0273 GTTGCTCCACAAATGACGGATACATGGGATAAACAGCTGGCAAGCTATGTTTCATCAAGC

O-COI_0482 GTTGCTCCACAAATGACGGATACATGGGATAAACAGCTGGCAAGCTATGTTTCATCAAGT

DNA Homology ***********************************************************

A1-MHA_0255 V A P Q M T D T W D K Q L A S Y V S S S

B-COK_0273 V A P Q M T D T W D K Q L A S Y V S S S

O-COI_0482 V A P Q M T D T W D K Q L A S Y V S S S

Protein Homology * * * * * * * * * * * * * * * * * * * *

A1-MHA_0255 TTCCGTGTCACCGTATTAGCAACCATTGGGCAACAAGGTGTACAACTTATTCAAAAAACC

B-COK_0273 TTCCGTGTCACCGTATTAGCAACCATTGGGCAACAAGGTGTACAACTTATTCAAAAAACC

O-COI_0482 TTCCGTGTCACCGTATTAGCAACCATTGGGCAACAAGGTGTACAACTTATTCAAAAAACC

DNA Homology ************************************************************

A1-MHA_0255 F R V T V L A T I G Q Q G V Q L I Q K T

B-COK_0273 F R V T V L A T I G Q Q G V Q L I Q K T

O-COI_0482 F R V T V L A T I G Q Q G V Q L I Q K T

Protein Homology * * * * * * * * * * * * * * * * * * * *

A1-MHA_0255 GTTATGGTGATTAACCTTTGGTTAGGGGCACACTTAGTTATTTCAGGCGATCTGAGTATT

B-COK_0273 GTTATGGTGATCAACCTTTGGCTAGGGGCACACTTAGTTATTTCAGGCGATCTTAGTATT

O-COI_0482 GTTATGGTGATTAACCTTTGGTTAGGGGCACACTTAGTTATTTCAGGCGATCTGAGTATT

DNA Homology *********** ********* ******************************* ******

A1-MHA_0255 V M V I N L W L G A H L V I S G D L S I

B-COK_0273 V M V I N L W L G A H L V I S G D L S I

O-COI_0482 V M V I N L W L G A H L V I S G D L S I

Protein Homology * * * * * * * * * * * * * * * * * * * *

A1-MHA_0255 GGGCAATTAATTGCCTTTAATATGCTATCAGGGCAAGTGATTGCACCGGTGATTCGGCTG

B-COK_0273 GGGCAATTAATTGCCTTTAATATGCTATCAGGGCAAGTGATTGCACCGGTGATTCGGCTG

O-COI_0482 GGGCAATTAATTGCCTTTAATATGCTATCAGGGCAAGTGATTGCACCGGTGATTCGGCTG

DNA Homology ************************************************************

A1-MHA_0255 G Q L I A F N M L S G Q V I A P V I R L

B-COK_0273 G Q L I A F N M L S G Q V I A P V I R L

O-COI_0482 G Q L I A F N M L S G Q V I A P V I R L

Protein Homology * * * * * * * * * * * * * * * * * * * *

A1-MHA_0255 GCTCAGCTCTGGCAAGATTTCCAACAAGTTGGGATTTCCGTCACTCGCTTAGGTGATGTT

B-COK_0273 GCTCAGCTCTGGCAAGATTTCCAACAAGTTGGGATTTCCGTCACTCGCTTAGGTGATGTT

O-COI_0482 GCTCAGCTCTGGCAAGATTTCCAACAAGTTGGGATTTCCGTCACTCGCTTAGGTGATGTT

DNA Homology ************************************************************

A1-MHA_0255 A Q L W Q D F Q Q V G I S V T R L G D V

B-COK_0273 A Q L W Q D F Q Q V G I S V T R L G D V

O-COI_0482 A Q L W Q D F Q Q V G I S V T R L G D V

Protein Homology * * * * * * * * * * * * * * * * * * * *

A1-MHA_0255 TTAAACTCTCCAACCGAACAATATCAAGGCAAATTATCACTACCAGAAATAAAAGGCGAT

B-COK_0273 TTAAACTCGCCAACCGAACAATATCAAGGCAAATTATCACTACCAGAAATAAAAGGCGAT

O-COI_0482 TTAAACTCTCCAACCGAACAATATCAAGGCAAATTATCACTACCAGAAATAAAAGGCGAT

DNA Homology ******** ***************************************************

A1-MHA_0255 L N S P T E Q Y Q G K L S L P E I K G D

B-COK_0273 L N S P T E Q Y Q G K L S L P E I K G D

O-COI_0482 L N S P T E Q Y Q G K L S L P E I K G D

Protein Homology * * * * * * * * * * * * * * * * * * * *

A1-MHA_0255 ATCTCATTTAAAAATATCCGCTTTAGATATAAACCAGATGCACCAACTATTTTAAATAAT

B-COK_0273 ATCTCATTTAAAAATATCCGCTTTAGATATAAACCAGATGCACCAACTATTTTAAATAAT

O-COI_0482 ATCTCATTTAAAAATATCCGCTTTAGATATAAACCAGATGCACCAACTATTTTAAATAAT

DNA Homology ************************************************************

A1-MHA_0255 I S F K N I R F R Y K P D A P T I L N N

B-COK_0273 I S F K N I R F R Y K P D A P T I L N N

O-COI_0482 I S F K N I R F R Y K P D A P T I L N N

Protein Homology * * * * * * * * * * * * * * * * * * * *

A1-MHA_0255 GTGAATTTAGAAATTAGGCAAGGAGAAGTGATTGGGATTGTTGGACGTTCCGGTTCAGGC

B-COK_0273 GTGAATTTAGAAATTAGGCAAGGAGAAGTGATTGGGATTGTTGGACGTTCCGGTTCAGGC

O-COI_0482 GTGAATTTAGAAATTAGGCAAGGAGAAGTGATTGGGATTGTTGGACGTTCCGGTTCAGGC

DNA Homology ************************************************************

A1-MHA_0255 V N L E I R Q G E V I G I V G R S G S G

B-COK_0273 V N L E I R Q G E V I G I V G R S G S G

O-COI_0482 V N L E I R Q G E V I G I V G R S G S G

Protein Homology * * * * * * * * * * * * * * * * * * * *

A1-MHA_0255 AAAAGTACTCTGACTAAATTACTGCAACGTTTTTATATTCCTGAAAATGGGCAGGTTTTG

B-COK_0273 AAAAGTACTCTGACTAAATTACTGCAACGTTTTTATATTCCTGAAAATGGGCAGGTTTTG

O-COI_0482 AAAAGTACTCTGACTAAATTACTGCAACGTTTTTATATTCCTGAAAATGGGCAGGTTTTG

DNA Homology ************************************************************

A1-MHA_0255 K S T L T K L L Q R F Y I P E N G Q V L

B-COK_0273 K S T L T K L L Q R F Y I P E N G Q V L

O-COI_0482 K S T L T K L L Q R F Y I P E N G Q V L

Protein Homology * * * * * * * * * * * * * * * * * * * *

A1-MHA_0255 ATTGATGGACATGATCTAGCCTTAGCTGATCCAAACTGGCTACGCCGTCAAATAGGTGTA

B-COK_0273 ATTGATGGACATGATCTAGCCTTAGCTGATCCAAACTGGCTACGCCGTCAAATAGGTGTA

O-COI_0482 ATTGATGGACATGATCTAGCCTTAGCTGATCCAAACTGGCTACGCCGTCAAATAGGTGTA

DNA Homology ************************************************************

A1-MHA_0255 I D G H D L A L A D P N W L R R Q I G V

B-COK_0273 I D G H D L A L A D P N W L R R Q I G V

O-COI_0482 I D G H D L A L A D P N W L R R Q I G V

Protein Homology * * * * * * * * * * * * * * * * * * * *

A1-MHA_0255 GTGCTGCAAGATAATGTGTTATTAAACCGCAGTATCCGAGAAAATATTGCGCTATCAGAT

B-COK_0273 GTGCTGCAAGATAATGTGTTATTAAACCGCAGTATCCGAGAAAATATTGCGCTATCAGAT

O-COI_0482 GTGCTGCAAGATAATGTGTTATTAAACCGCAGTATCCGAGAAAATATTGCGCTATCAGAT

DNA Homology ************************************************************

A1-MHA_0255 V L Q D N V L L N R S I R E N I A L S D

B-COK_0273 V L Q D N V L L N R S I R E N I A L S D

O-COI_0482 V L Q D N V L L N R S I R E N I A L S D

Protein Homology * * * * * * * * * * * * * * * * * * * *

A1-MHA_0255 CCAGGAATGCCAATGGAGCGAGTAATTTATGCAGCAAAATTAGCAGGGGCTCACGATTTT

B-COK_0273 CCAGGAATGCCAATGGAGCGAGTAATTTATGCAGCAAAATTAGCAGGGGCTCACGATTTT

O-COI_0482 CCAGGAATGCCAATGGAGCGAGTAATTTATGCAGCAAAATTAGCAGGGGCTCACGATTTT

DNA Homology ************************************************************

A1-MHA_0255 P G M P M E R V I Y A A K L A G A H D F

B-COK_0273 P G M P M E R V I Y A A K L A G A H D F

O-COI_0482 P G M P M E R V I Y A A K L A G A H D F

Protein Homology * * * * * * * * * * * * * * * * * * * *

A1-MHA_0255 ATTTCAGAATTGCGTGAAGGTTATAACACCATTGTGGGTGAACAAGGAGCGGGGCTTTCA

B-COK_0273 ATTTCAGAATTGCGTGAAGGTTATAACACCATTGTGGGTGAACAAGGAGCGGGGCTTTCA

O-COI_0482 ATTTCAGAATTGCGTGAAGGTTATAACACCATTGTGGGTGAACAAGGAGCGGGGCTTTCA

DNA Homology ************************************************************

A1-MHA_0255 I S E L R E G Y N T I V G E Q G A G L S

B-COK_0273 I S E L R E G Y N T I V G E Q G A G L S

O-COI_0482 I S E L R E G Y N T I V G E Q G A G L S

Protein Homology * * * * * * * * * * * * * * * * * * * *

A1-MHA_0255 GGCGGGCAACGCCAACGGATTGCGATTGCTCGAGCTTTGGTAAACAACCCGAAAATCCTG

B-COK_0273 GGCGGGCAACGCCAACGGATTGCGATTGCTCGAGCTTTGGTAAACAACCCGAAAATCTTG

O-COI_0482 GGCGGGCAACGCCAACGGATTGCGATTGCTCGAGCTTTGGTAAACAACCCGAAAATCCTG

DNA Homology ********************************************************* **

A1-MHA_0255 G G Q R Q R I A I A R A L V N N P K I L

B-COK_0273 G G Q R Q R I A I A R A L V N N P K I L

O-COI_0482 G G Q R Q R I A I A R A L V N N P K I L

Protein Homology * * * * * * * * * * * * * * * * * * * *

A1-MHA_0255 ATTTTTGATGAGGCAACCAGTGCCCTCGATTACGAATCTGAGCATATTATTATGCAAAAT

B-COK_0273 ATTTTTGATGAGGCAACCAGTGCCCTCGATTACGAATCTGAGCATATTATTATGCAAAAT

O-COI_0482 ATTTTTGATGAGGCAACCAGTGCCCTCGATTACGAATCTGAGCATATTATTATGCAAAAT

DNA Homology ************************************************************

A1-MHA_0255 I F D E A T S A L D Y E S E H I I M Q N

B-COK_0273 I F D E A T S A L D Y E S E H I I M Q N

O-COI_0482 I F D E A T S A L D Y E S E H I I M Q N

Protein Homology * * * * * * * * * * * * * * * * * * * *

A1-MHA_0255 ATGCAAAAAATATGCCAAGGCAGAACCGTGATTTTGATTGCACATCGTTTATCGACCGTC

B-COK_0273 ATGCAAAAAATATGCCAAGGCAGAACCGTGATTTTGATTGCACATCGTTTATCGACCGTC

O-COI_0482 ATGCAAAAAATATGCCAAGACAGAACCGTGATTTTGATTGCACATCGTTTATCGACCGTC

DNA Homology ******************* ****************************************

A1-MHA_0255 M Q K I C Q G R T V I L I A H R L S T V

B-COK_0273 M Q K I C Q G R T V I L I A H R L S T V

O-COI_0482 M Q K I C Q D R T V I L I A H R L S T V

Protein Homology * * * * * * . * * * * * * * * * * * * *

A1-MHA_0255 AAAAATGCGGATCGAATTATTGTGATGGAAAAGGGGGAAATTGTTGAGCAAGGCAAGCAC

B-COK_0273 AAAAATGCGGATCGAATTATTGTGATGGAAAAGGGGGAAATTGTTGAGCAAGGCAAGCAC

O-COI_0482 AAAAATGCGGATCGAATTATTGTGATGGAAAAGGGGGAAATTGTTGAGCAAGGCAAGCAC

DNA Homology ************************************************************

A1-MHA_0255 K N A D R I I V M E K G E I V E Q G K H

B-COK_0273 K N A D R I I V M E K G E I V E Q G K H

O-COI_0482 K N A D R I I V M E K G E I V E Q G K H

Protein Homology * * * * * * * * * * * * * * * * * * * *

A1-MHA_0255 CACGAATTACTGCAAAACAGTAACGGACTTTATTCCTACTTACACCAATTACAACTTAAT

B-COK_0273 CACGAATTACTGCAAAACAGTAACGGACTTTATTCCTACTTACACCAATTACAACTTAAT

O-COI_0482 CACGAATTACTGCAAAACAGTAACGGACTTTATTCCTACTTACACCAATTACAACTTAAT

DNA Homology ************************************************************

A1-MHA_0255 H E L L Q N S N G L Y S Y L H Q L Q L N

B-COK_0273 H E L L Q N S N G L Y S Y L H Q L Q L N

O-COI_0482 H E L L Q N S N G L Y S Y L H Q L Q L N

Protein Homology * * * * * * * * * * * * * * * * * * * *
