# Supplementary material for: A three-way comparative genomic analysis of Mannheimia haemolytica isolates
Source: BMC Genomics. 2010 Oct 4;11:535. doi: 10.1186/1471-2164-11-535 (PMC3091684; doi:10.1186/1471-2164-11-535)
Supplement: Additional file 9 — Table S9: M. haemolytica LPS and other complex carbohydrate synthesis components. [file 1471-2164-11-535-S9.DOC]

**Table S9:** *M. haemolytica* LPS and other complex carbohydrate synthesis components

| **A1 Gene ID** | **B Gene ID** | **O Gene ID** | **Product** | **Component** | **% identity** |
| --- | --- | --- | --- | --- | --- |
| MHA_0068 | COK_0785 | COI_1882 | 3-deoxy-D-manno-octulosonic-acid transferase | Lipid A/core | 99.95 |
| MHA_0100 | COK_0753 | COI_1850 | possible glycosyltransferase | Exopolysaccharide | 99.83 |
| MHA_0101 | COK_0752 | COI_1849 | possible glycosyltransferase | Exopolysaccharide | 99.79 |
| MHA_0102 | COK_0751 | COI_1848 | hypothetical protein MHA_0102 |  | 100 |
| MHA_0103 | COK_0750 | COI_1847 | possible glycosyltransferase |  | 99.83 |
| MHA_0104 | COK_0748COK_0749 | COI_1846 | possible alpha-2,3-sialyltransferase |  | 99.60 |
| MHA_0105 | COK_0747 | COI_1845 | PST family polysaccharide transporter | Exopolysaccharide | 99.78 |
| MHA_0106 | COK_0746 | COI_1844 | UDP-N-acetylglucosamine 2-epimerase | Exopolysaccharide | 99.83 |
| MHA_0191 | COK_1758 | COI_2338 | heptosyltransferase II (inner core) | Lipid A/core | 99.94 |
| MHA_0517 | COK_0614 | COI_0041 | phosphomannomutase | GDP-mannose pathway | 99.68 |
| MHA_0521 | 0 | 0 | UDP-N-acetylglucosamine 2-epimerase | Exopolysaccharide | NA |
| MHA_0522 | COK_0352 | COI_1083 | ECA biosynthesis protein WecC | Exopolysaccharide | 75.04 |
| MHA_0726 | COK_0354 | COI_1085 | UDP-N-acetyl muramyl pentapeptide phosphotransferase | Exopolysaccharide | 100 |
| MHA_0727 | COK_0353 | COI_1084 | lipopolysaccharide chain length determinant protein | Saccharide processing | 100 |
| MHA_0728 | COK_0352 | COI_1083 | UDP-N-acetyl-D-mannosaminuronic acid dehydrogenase WecC | Exopolysaccharide | 99.84 |
| MHA_0729 | COK_0351 | COI_1082 | possible fucosamine acetyl transferase |  | 99.79 |
| MHA_0730 | COK_0350 | COI_1081 | ECA biosynthesis protein WecE | Exopolysaccharide | 99.65 |
| MHA_0825 | COK_0960 | COI_0993 | lipopolysaccharide N-acetylglucosaminyltransferase | Lipid A/core | 99.94 |
| MHA_1050 | COK_0398 | COI_1984 | ADP-glyceromanno-heptose 6-epimerase | Lipid A/core | 100 |
| MHA_1349 | COK_2454 | COI_1924 | UDP-glucose 4-epimerase | UDP-galactose pathway | 99.87 |
| MHA_1392 | COK_0006 | COI_0716 | lipid A acyltransferase | Lipid A/core | 99.86 |
| MHA_1558 | COK_2066 | COI_0127 | glycosyltransferase LpsA | Lipid A/core | 78.88 |
| MHA_1562 | COK_2072 | COI_0133 | lipid A acyltransferase | Lipid A/core | 99.86 |
| MHA_1599 | COK_2107 | COI_0168 | possible heptosyltransferase II (inner core) | Lipid A/core | 100 |
| MHA_1600 | COK_2108 | COI_0169 | possible heptosyltransferase II (inner core) | Lipid A/core | 99.94 |
| MHA_1845 | COK_1319COK_1320 | COI_2468COI_2469 | possible O-antigen ligase | Lipid A/core | 83.00 |
| MHA_1846 | COK_1321 | COI_2467 | dTDP-glucose 4,6-dehydratase | L-rhamnose pathway | 80.17 |
| MHA_1847 | 0 | 0 | hypothetical protein MHA_1847 |  | NA |
| MHA_1848 | COK_1322 | COI_2466 | possible LPS sugar transferase |  | 68.03 |
| MHA_1849 | 0 | 0 | probable glycosyltranferase |  | NA |
| MHA_1850 | 0 | 0 | possible glycosyltransferase |  | NA |
| MHA_1851 | 0 | 0 | hypothetical protein MHA_1851 |  | NA |
| MHA_1852 | 0 | 0 | possible sialyltransferase |  | NA |
| MHA_1853 | COK_1325 | COI_2463 | O-antigen chain length determining protein | Saccharide processing | 98.71 |
| MHA_2240 | COK_1306 | COI_2482 | phosphomannomutase | GDP-mannose pathway | 99.95 |
| MHA_2564 | COK_2219 | COI_0826 | possible bifunctional D,D-heptose 1-phosphate adenosyltransferase/7-phosphate kinase | Lipid A/core | 99.81 |
| MHA_2704 | COK_1106 | COI_1632 | heptosyltransferase II (inner core) | Lipid A/core | 99.54 |
| MHA_2705 | COK_1104COK_1105 | COI_1634COI_1633 | heptosyltransferase I (inner core) | Lipid A/core | 99.78 |
